# Diagnostic yield of pediatric and prenatal exome sequencing in a diverse population

**DOI:** 10.1038/s41525-023-00353-0

**Published:** 2023-05-26

**Authors:** Anne Slavotinek, Shannon Rego, Nuriye Sahin-Hodoglugil, Mark Kvale, Billie Lianoglou, Tiffany Yip, Hannah Hoban, Simon Outram, Beatrice Anguiano, Flavia Chen, Jeremy Michelson, Roberta M. Cilio, Cynthia Curry, Renata C. Gallagher, Marisa Gardner, Rachel Kuperman, Bryce Mendelsohn, Elliott Sherr, Joseph Shieh, Jonathan Strober, Allison Tam, Jessica Tenney, William Weiss, Amy Whittle, Garrett Chin, Amanda Faubel, Hannah Prasad, Yusuph Mavura, Jessica Van Ziffle, W. Patrick Devine, Ugur Hodoglugil, Pierre-Marie Martin, Teresa N. Sparks, Barbara Koenig, Sara Ackerman, Neil Risch, Pui-Yan Kwok, Mary E. Norton

**Affiliations:** 1 Department of Pediatrics, University of California, San Francisco, San Francisco, CA USA; 2 Institute for Human Genetics, University of California San Francisco, San Francisco, CA USA; 3 Department of Epidemiology & Biostatistics, University of California San Francisco, San Francisco, CA USA; 4 Department of Surgery, University of California, San Francisco, San Francisco, CA USA; 5 Institute for Health & Aging, School of Nursing, University of California San Francisco, San Francisco, CA USA; 6 Institute of Human Nutrition, Columbia University Medical Center, New York, NY USA; 7 Division of Pediatric Neurology, Department of Pediatrics, University of Louvain, Brussels, Belgium; 8 Genetic Medicine, University of California, San Francisco, Fresno, CA USA; 9 Department of Neurology, UCSF Benioff Children’s Hospital Oakland, Oakland, CA USA; 10 Eysz, Inc, Piedmont, CA USA; 11 Division of Genetics, Kaiser Permanente Oakland Medical Center, Oakland, CA USA; 12 Division of Child Neurology, Department of Neurology, University of California, San Francisco, San Francisco, CA USA; 13 Division of Child Neurology, Zuckerberg San Francisco General Hospital, San Francisco, San Francisco, CA USA; 14 Division of Pediatrics, Zuckerberg San Francisco General Hospital, San Francisco, San Francisco, CA USA; 15 Department of Pathology, University of California San Francisco, San Francisco, CA USA; 16 Genomic Medicine Laboratory, University of California San Francisco, San Francisco, CA USA; 17 Division of Maternal Fetal Medicine, Department of Obstetrics, Gynecology, and Reproductive Sciences, San Francisco, USA; 18 Program in Bioethics, University of California, San Francisco, San Francisco, CA USA; 19 Department of Social & Behavioral Sciences, School of Nursing, University of California San Francisco, San Francisco, CA USA; 20 Cardiovascular Research Institute, University of California San Francisco, San Francisco, CA USA

**Keywords:** Neurological disorders, Molecular medicine, Genetic testing

## Abstract

The diagnostic yield of exome sequencing (ES) has primarily been evaluated in individuals of European ancestry, with less focus on underrepresented minority (URM) and underserved (US) patients. We evaluated the diagnostic yield of ES in a cohort of predominantly US and URM pediatric and prenatal patients suspected to have a genetic disorder. Eligible pediatric patients had multiple congenital anomalies and/or neurocognitive disabilities and prenatal patients had one or more structural anomalies, disorders of fetal growth, or fetal effusions. URM and US patients were prioritized for enrollment and underwent ES at a single academic center. We identified definitive positive or probable positive results in 201/845 (23.8%) patients, with a significantly higher diagnostic rate in pediatric (26.7%) compared to prenatal patients (19.0%) (*P* = 0.01). For both pediatric and prenatal patients, the diagnostic yield and frequency of inconclusive findings did not differ significantly between URM and non-URM patients or between patients with US status and those without US status. Our results demonstrate a similar diagnostic yield of ES between prenatal and pediatric URM/US patients and non-URM/US patients for positive and inconclusive results. These data support the use of ES to identify clinically relevant variants in patients from diverse populations.

## Introduction

The global prevalence of congenital disorders that are life-limiting or cause lifelong impairment is estimated at 5% to 7%^1–3^. About half of these congenital disorders are attributable to variants in single genes that are amenable to detection by genetic testing^4^. Exome sequencing (ES) enables the simultaneous evaluation of numerous genes for variants that cause Mendelian disorders, potentially facilitating early diagnosis and the implementation of targeted therapies so that patient outcomes can be improved. The diagnostic yield from ES was reported as 36% from one meta-analysis examining the use of this test for clinical indications including developmental delays (DD), intellectual disability (ID), and multiple congenital anomalies (MCAs)^5^. This promising yield led to a consensus statement that recommends ES as the first-line genetic test for pediatric and adult patients with clinical findings that fall within these categories^5^. ES has also been used in prenatal cohorts, primarily in the setting of structural fetal anomalies^6–8^. The diagnostic yield in these prenatal cohorts is lower, at 8.5–10%, possibly due to differences in test indications, case interpretation, and limitations of prenatal phenotyping. For this reason, insurance coverage and professional recommendations for ES in prenatal cases have lagged, reflecting differences in the perceived costs and benefits of the test^9,10^.

The diagnostic yield and clinical utility of ES have primarily been evaluated in non-Hispanic white patients and families who are typically well served medically^11–15^. Relatively little attention has been paid to diagnostic yield and clinical utility in diverse populations, including underserved (US) and underrepresented minority (URM) patients in the United States^16^. Concerns have been raised that equitable inclusion of patients from diverse populations in research linking genes and disease has not yet been achieved^17–20^. Without efforts directed at addressing this gap in genetic testing research, disparities in access to and implementation of ES and genome sequencing (GS) may be further exacerbated^16^. Studies that prioritize US and URM participants are critical to involving these populations in genetic testing and for optimal use of genomic technologies^21^.

The Program in Prenatal and Pediatric Genomic Sequencing (P^3^EGS) at the University of California, San Francisco (UCSF), is part of the Clinical Sequencing Evidence-generating Research (CSER) consortium^22^. CSER’s second phase has been directed towards a study of clinical utility when ES and GS are integrated into the clinical care of patients, including US and URM patients. In the P^3^EGS study, our objective was to perform ES as a clinical test for the fetuses of pregnant patients and pediatric patients in whom a genetic etiology was suspected based on clinical findings, and prior genetic testing with microarray, single-gene or gene panel sequencing had failed to yield a diagnosis.

## Results

### Individuals studied and demographics

A total of 845 patients, comprising 529 pediatric and 316 prenatal probands, were enrolled (Table 1). The pediatric group included more males (290/529, 54.8%) than females (239/529, 45.2%), which was significantly different from a 50:50 distribution (χ^2^ = 4.92, *P* = 0.027). The prenatal group also included more male (171/316, 54.1%) than female fetuses (145/316, 45.9%); a difference that was not significant (χ^2^ = 2.14, *P* = 0.144). In all, 86/529 (16.3%) of pediatric patients were younger than one year of age and 405/529 (76.6%) were ten years of age or younger at enrollment (Supplementary Table 1), reflecting the early onset of clinical findings associated with neurodevelopmental disorders and the enrollment categories used in this study. In the prenatal cohort, the mean gestational age at enrollment was 23.5 weeks. Overall, 135/316 (42.7%) of pregnancies were terminated and 122 (38.0%) resulted in a living child that survived the neonatal period (Supplementary Table 2). For the remaining pregnancies, 19 (6.4%) resulted in a stillbirth at ≥20 weeks gestation, 7 (2.2%) in a miscarriage at <20 weeks, and 33 (10.5%) in a neonatal death. Of the patients choosing pregnancy termination, 92.6% received ES results after the conclusion of the pregnancy.

**Table 1:** Participant demographics in the program in prenatal and pediatric genomic sequencing (P^3^EGS) study.

	Pediatric (*n* = 529) *n* (%)	Prenatal (*n* = 316) *n* (%)
Sex of proband		
Female	239 (45.2)	145 (45.5)
Male	290 (54.8)	171 (54.5)
Median proband/gestational age (range)	5.0 years (0–25)	23.5 weeks (13–39)
Median maternal age at child’s conception in years (range)	28.2 (15–46)	33.1 (20–56)
Median paternal age at child’s conception in years (range)	32.2 (18–73)	35.0 (21–68)
Maternal race/ethnicity		
American Indian, Native American, Alaska Native	6 (1.1)	0 (0)
Asian	57 (10.8)	49 (15.5)
Black/African American	21 (4.0)	3 (0.9)
Native Hawaiian/Pacific Islander	4 (0.8)	0 (0)
White/European American	96 (18.1)	115 (36.4)
Middle Eastern or North African/Mediterranean	12 (2.3)	5 (1.6)
Hispanic/Latino(a)	228 (43.1)	49 (15.5)
More than one race/ethnicity	41 (7.8)	32 (10.1)
Unknown, None of the above	64 (12.1)	63 (19.9)
Paternal race/ethnicity		
American Indian, Native American, Alaska Native	6 (1.1)	0 (0)
Asian	50 (9.5)	43 (14.4)
Black/African American	20 (3.8)	3 (0.8)
Native Hawaiian/Pacific Islander	5 (0.9)	0 (0)
White/European American	101 (19.1)	115 (38.4)
Middle Eastern or North African/Mediterranean	12 (2.3)	5 (1.9)
Hispanic/Latino(a)	203 (38.4)	49 (16.7)
More than one race/ethnicity	33 (6.2)	25 (7.1)
Unknown, None of the above	99 (18.7)	76 (14.4)
URM proband by race/ethnicity of either parent	397 (85.7)	157 (63.8)
Underserved status – Total	457 (86.4)	146 (46.2)
US status (Medi-Cal, Medicaid or no insurance)	432 (81.7)	73 (23.6)
MUA/P	148 (28.0)	74 (23.4)
HPSA	144 (27.2)	78 (24.7)

*URM* Under-represented minority, *MUA/P* Medically Underserved Area/Population, *HPSA* Health Professional Shortage Area.

**Fig. 1:**
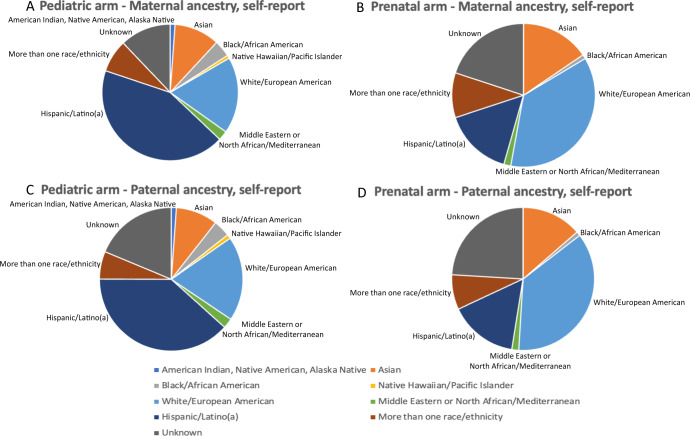
Distribution of ancestry in 845 patients enrolled in the Program in Prenatal and Prenatal Genomic Sequencing (P^3^EGS) study. Each chart shows the distribution of ancestry according to the arm of the study (Pediatric and Prenatal) and the sex of the participant. Ancestries depicted are American Indian, Native American (blue), Alaskan Native (Asian (orange), White/European (light blue), Middle Eastern/North African (green), Hispanic/Latino or Latina (dark blue), More than one race/ethnicity (brown), Unknown, none of the above (gray). **A** Pediatric patients, maternal ancestry. **B** Pediatric patients, paternal ancestry. **C** Prenatal patients, maternal ancestry. **D** Prenatal patients, paternal ancestry.

As proband ages varied at the time of enrollment, we tabulated mean parental ages at the time of the proband’s conception. The median maternal age at the time of proband conception was 28.2 years for pediatric and 33.1 years for prenatal patients. Median paternal age at the time of conception was 32.2 years for pediatric and 35.0 years for prenatal patients (Table 1). Overall, 554/845 (65.6%) pediatric and prenatal patients had at least one parent who self-identified as URM, 155/845 (18.3%) were non-URM (i.e., both parents white/European), and in 136/845 (16.1%) the race/ethnicity was unknown or missing for both parents or one parent while the other self-reported white/European (Table 1; Fig. 1A–D). There were more URM families among the pediatric patients (397/463, 85.7%) compared to the prenatal cohort (157/246, 63.8%). The largest race/ethnicity group among the parents of pediatric cases was Hispanic/Latino (43.1% of mothers, 38.4% of fathers), followed by white/European (18.1% of mothers, 19.1% of fathers). The largest race/ethnicity group among the parents of the prenatal cases was white/European (36.4% of mothers, 38.4% of fathers), followed by Hispanic/Latino (15.5% of mothers, 16.7% of fathers; Table 1).

In the entire cohort, 505/845 patients (59.8%) had public insurance through Medi-Cal or had no insurance coverage. Public insurance was more frequent for pediatric (432/529, 81.7%) compared to prenatal patients (73/316, 23.1%). In addition, 148/529 (28.0%) pediatric patients and 74/316 (23.4%) prenatal patients and mothers were domiciled at an address and zip code that defined a medically underserved area/population (MUA/P) and 144/529 (27.2%) pediatric patients and 78/316 (24.7%) prenatal patients and mothers were domiciled at an address and zip code defined as a health provider shortage area (HPSA). In total, 457/529 (86.4%) pediatric patients and 146/316 (46.2%) prenatal patients met at least one of the US categories.

### Diagnostic categories

For the pediatric cohort, the most common diagnostic category was MCA with ID (252/529, 47.6%), followed by MCA without ID (105/529, 19.8%) and ID only (48/529, 9.1%; Table 2). Enrollment in the categories of metabolic disease without ID, epilepsy without ID and neurodegenerative disease/cerebral palsy (CP) with or without ID were relatively low and this may reflect consultation patterns, including a greater emphasis on referrals for genetic testing for patients with ID, in addition to the relative frequencies of these phenotypes and the availabilty of diagnostic panels for genetic testing in addition to ES. The prenatal cohort included 125/316 pregnancies with a single sonographic anomaly (39.6%) and 191/316 with multiple structural anomalies (60.4%, Table 2).

**Table 2:** Exome sequencing results of P^3^EGS patients (based on pediatric inclusion criteria and prenatal phenotypes with ultrasound).

	Definitive/probable positive *n* (%^a^)	Inconclusive *n* (%^a^)	Negative *n* (%^a^)	Total *n* (%^b^)
Pediatric inclusion criteria (*n* = 529)				
ID + MCA	71 (28.2)	31 (12.3)	150 (59.5)	252 (47.6)
MCA	29 (27.6)	16 (15.2)	60 (57.1)	105 (19.8)
ID only	8 (16.7)	6 (12.5)	34 (70.8)	48 (9.1)
Epilepsy + ID	9 (21.4)	8 (19.0)	25 (59.5)	42 (7.9)
NDD + ID	9 (29.0)	5 (16.1)	17 (54.8)	31 (5.9)
Metabolic disease, no ID	—	3 (25.0)	9 (75.0)	12 (2.3)
NDD, no ID	4 (40.0)	1 (10.0)	5 (50.0)	10 (1.9)
Metabolic disease + ID	4 (44.4)	3 (33.3)	2 (22.2)	9 (1.7)
Encephalopathy	2 (40.0)	—	3 (60.0)	5 (0.9)
Epilepsy no ID	1 (33.3)	—	2 (66.7)	3 (0.6)
Other^c^	4 (33.3)	1 (8.3)	7 (58.3)	12 (2.3)
Pediatric patients total	141 (26.7)	74 (14.0)	314 (59.4)	529 (100.0)
Prenatal inclusion criteria by ultrasound findings at enrollment (*n* = 316)				
Isolated anomaly	15 (12.0)	6 (4.8)	104 (83.2)	125 (39.6)
Cardiovascular	3 (11.5)	3 (11.5)	20 (76.9)	26 (8.2)
Central nervous system	4 (16.0)	0 (0)	21 (84.0)	25 (7.9)
Neck	1 (5.3)	2 (10.5)	16 (84.2)	19 (6.0)
Effusions	2 (12.5)	—	14 (87.5)	16 (5.1)
Gastrointestinal tract	—	1 (6.7)	14 (93.3)	15 (4.7)
Skeletal	2 (20.0)	—	8 (80.0)	10 (3.2)
Genitourinary Tract	2 (28.6)	—	5 (71.4)	7 (2.2)
Face	1 (50.0)	—	1 (50.0)	2 (0.6)
Spine	—	—	2 (100.0)	2 (0.6)
Abdominal wall	—	—	1 (100.0)	1 (0.3)
Fetal growth alteration	—	—	1 (100.0)	1 (0.3)
Hematologic/lymphatic/skin	—	—	1 (100.0)	1 (0.3)
Ear	—	—	—	—
Pulmonary	—	—	—	—
Multiple anomalies	45 (23.6)	14 (7.3)	132 (69.1)	191 (60.4)
Prenatal patients total	60 (19.0)	20 (6.3)	236 (74.7)	316 (100.0)

^a^Row %; ^b^Column %, *ID* Intellectual Disability, *MCA* Multiple Congenital Anomalies, *NDD* Neurodegenerative disorder/Cerebral palsy; Other^c^ Clinical findings associated with patients enrolled under ‘Other’ are provided with Table S12.

### Exome sequencing

Trio ES was performed in 583 (69.0%) cases, 122 (14.4%) patients were sequenced as duos, most commonly including the mother and child or fetus, and 109 (12.9%) patients were sequenced with a proband first approach (Supplementary Tables 3 and 4). Trio ES was more frequent in prenatal (257/316, 81.3%) compared to pediatric (326/529, 61.6%) patients and there was a higher prevalence of duo sequencing in pediatric (108/529, 20.4%) compared to prenatal (14/316, 4.4%) patients, reflecting a greater availability of fathers for prenatal versus pediatric patients in our study.

### Diagnostic yield—pediatric versus prenatal

Overall, 201/845 (23.8%) patients received a positive (defined here as definitive positive or probable positive) diagnosis (Table 3). Of these, 137 (68.2%) had a pathogenic (P) or likely pathogenic (LP) variant in a gene with autosomal dominant (AD) inheritance, 40 (19.9%) had two P or LP variants or one P/LP variant and one variant of unknown significance (VUS) in a gene with autosomal recessive (AR) inheritance, and 24 (11.9%) had a P or LP variant in a gene with X-linked (XL) inheritance. In addition, 94/845 (11.1%) patients had inconclusive results (most had at least one VUS), and 550/845 (65.1%) received a negative result. The overwhelming majority of VUSs in our study (95%) were due to variant uncertainty, as opposed to gene uncertainty. The diagnostic yield was higher in pediatric patients, with 141/529 (26.7%) receiving a positive diagnosis compared to 60/316 (19.0%) in the prenatal group (χ^2^ = 6.41, *P* = 0.01). The frequency of inconclusive results was also significantly greater in pediatric (74/529, 14.0%) compared to prenatal patients (20/316, 6.3%; χ^2^ = 11.74, *P* = 0.0006). Of interest, the greater diagnostic yield in the pediatric cases was largely attributable to pathogenic variants in genes with AD inheritance (98/529 = 18.5% for pediatric cases versus 39/316 = 12.3% for the prenatal cases), including variants that were de novo, inherited from a parent, or of unknown segregation. The proportion of positive versus inconclusive cases also differed by mode of inheritance. For the pediatric cases, 69.5% of the positive results were for variants with AD inheritance versus 17.7% for AR inheritance, a ratio of 3.9:1. However, among the inconclusive results in pediatric patients, 40.5% were in variants with AD inheritance, while 44.6% were in AR genes, a ratio close to 1:1. This difference in mode of inheritance between positive and inconclusive results was highly statistically significant (χ^2^ = 19.95, *P* = 8.0 × 10^−6^). This trend was not observed among the prenatal group and for positive results, the ratio of AD to AR inheritance was 2.6:1 and among the inconclusive cases, the ratio was 2.0:1.

**Table 3:** Diagnostic yield and inconclusive rate by mode of inheritance.

Result type	Mode of inheritance	Pediatric (*n* = 529)	Prenatal (*n* = 316)
Definite/Probable Positive	AD^1^, de novo	67 (12.7%)	34 (10.8)
	AD, inherited	14 (2.6%)	4 (1.3%)
	AD, segregation unknown	17 (3.2%)	1 (0.3%)
	AR^2^, homozygous	14 (2.6%)	4 (1.3%)
	AR, compound heterozygous	11 (2.1%)	11 (3.5%)
	X-linked	18 (3.4%)	6 (1.9%)
	All	141 (26.7%)	60 (19.0%)
Inconclusive			
	AD, de novo	11 (2.1%)	6 (1.9%)
	AD, inherited	11 (2.1%)	4 (1.3%)
	AD, segregation unknown	8 (1.5%)	2 (0.6%)
	AR, homozygous	24 (4.5%)	3 (0.9%)
	AR, compound heterozygous	9 (1.7%)	3 (0.9%)
	X-linked	11 (2.1%)	2 (0.6%)
	All	74 (14.0%)	20 (6.3%)
Negative		314 (59.4%)	236 (74.7%)

*AD*^*1*^ autosomal dominant, *AR*^2^ autosomal recessive.

### Diagnostic yield by indication

There was no difference in the diagnostic yield by indication in the pediatric patients (Table 2) and although diagnostic yield for isolated ID was lower (8/48, 16.7%) than for ID with multiple congenital anomalies (71/252, 28.2%), this difference was not significant (χ^2^ = 2.82, *P* = 0.09). In the prenatal cases, those with multiple sonographic abnormalities were more likely to have a positive result (45/191, 23.6%) compared to prenatal cases with a single structural anomaly (15/125, 12.0%; χ^2^ = 6.56, *P* = 0.01). Interestingly, while the diagnostic yield of pediatric cases was overall higher than of prenatal, in pediatric and prenatal patients with multiple anomalies as a clinical indication for ES, the diagnostic yield was similar; with 100/357 (28.9%) positive pediatric cases compared to 45/191 (23.6%) in the prenatal group (χ^2^ = 1.22, *P* = 0.27).

### Diagnostic yield by number of family members sequenced

**Fig. 2:**
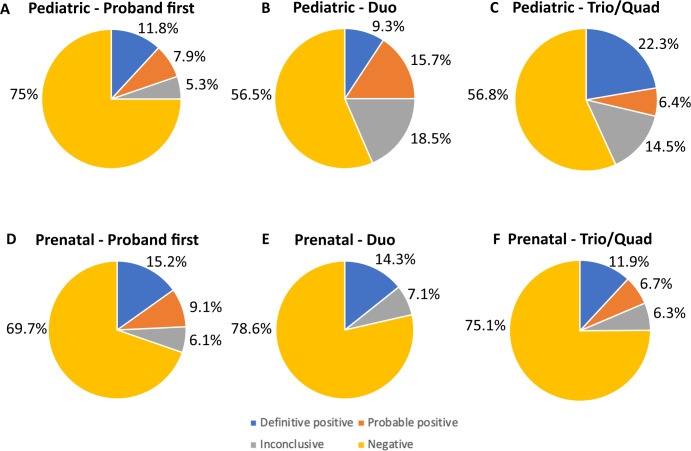
Diagnostic yield by sequencing approach in 845 patients enrolled in the Program in Prenatal and Prenatal Genomic Sequencing (P^3^EGS) study. The percentages of definitive positive (orange), probable positive (yellow), inconclusive (green) and negative (brown) results are shown for proband first, duo and trio sequencing approaches. There was no statistically significant difference in diagnostic yield with any sequencing approach. **A** Diagnostic yield with ‘proband first’ sequencing in pediatric patients. **B** Diagnostic yield with duo sequencing in pediatric patients. **C** Diagnostic yield with trio sequencing in pediatric patients. **D** Diagnostic yield with ‘proband first’ sequencing in prenatal patients. **E** Diagnostic yield with duo sequencing in prenatal patients. **F** Diagnostic yield with trio sequencing in prenatal patients.

Among the pediatric cases, there was a clear decrease in definitive positive diagnoses in the duo and proband first families (9.3% and 11.8%, respectively) compared to the quad and trio families (26.3% and 22.1% respectively; Supplementary Table 3; Fig. 2A–C). This was primarily observed with patients receiving P or LP results in genes with AD inheritance. In contrast, there was a higher rate of probable positive diagnoses among the duo families (15.7%), compared to the trio (6.7%) or quad families (0%; Supplementary Table 3). The higher rate of probable positive diagnoses was primarily due to variants in genes with AD inheritance when parental segregation of the variant could not be determined. There were fewer definitive positive and probable diagnoses in the ‘proband first’ families, with a yield of 19.7%, although this difference was not significantly different from trios (χ^2^ = 2.58, *P* = 0.108). In patients with variants in genes with AR inheritance, there was no overall difference in diagnostic yield by number of parents sequenced for both homozygotes and compound heterozygotes. Among the pediatric families, there was a higher rate of de novo, AD definitive and probable positive results in trios (17.5%) compared to the quad families (5.3%), although this increase was not significant (χ^2^ = 1.92, *P* = 0.17). There was a significant excess of positive, inherited AD variants (15.7%) in quads compared to trios (2.5%) (χ^2^ = 10.34, *P* = 0.0013).

In the prenatal families, there were no statistically significant differences in diagnostic yield based on the number of parents sequenced (16.7% for quads, 18.7% for trios, 14.3% for duos and 24.2% for proband first; Supplementary Table 4; Fig. 2D–F); however, the number of non-trio cases was small (59 total, or 18.7%), limiting power for comparisons. In the prenatal families, 39/60 (65.0%) of the definitive positive and probable positive results involved genes with AD inheritance, and 34/39 (87.2%) of these variants were de novo. In addition, 15/60 (25%) of definitive positive and probable positive results involved AR genes, while 6/60 (10.0%) involved XL genes.

### Diagnostic yield by age and sex of proband and prenatal outcome

Diagnostic yield for pediatric probands did not differ significantly by age of proband (Supplementary Table 1). Comparing positive diagnoses between affected male and female individuals (Supplementary Table 3), we noted a significantly higher diagnostic yield in female probands (77/239, 32.2%) compared to males (64/290, 22.1%) in pediatric families (*χ*^2^ = 6.41, *P* = 0.011). This difference was not observed in the prenatal families (Supplementary Table 4), with a female diagnostic yield of 26/145 (17.9%) and a male diagnostic yield of 34/171 (19.9%). Further examination of the pediatric probands by indication revealed that the sex difference was fully explained by probands with ID, with a diagnostic yield of 33.5% (56/167) for females with ID versus 20.8% (45/216) in males with ID, a statistically significant difference (*χ*^2^ = 7.82, *P* = 0.005; data not shown). There was no different in diagnostic yield in females without ID (28.2%; 20/71) versus males (26.7%;20/75, *χ*^2^ = 0.04, *P* = 0.84; data not shown). Diagnostic yield varied by pregnancy outcome (Supplementary Table 2). The diagnostic yield was significantly higher for pregnancies resulting in neonatal death (27.3%), pregnancy termination (27.4%) and miscarriage (28.6%) compared to pregnancies resulting in stillbirths (10.5%) or living children (8.2%, χ^2^ = 18.2, df = 4, *P* = 0.0011).

### Diagnostic yield by URM and US status

In the pediatric families, there was a similar diagnostic yield for URM (at least one parent URM) (26.0%) and non-URM (both parents not URM) individuals (27.7%; χ^2^ = 0.08, *P* = 0.78; Supplementary Table 5). There was no significant difference in yield based on the number of URM parents (25.1% for two URM parents versus 28.6% for one URM parent). The diagnostic rate was also not significantly different for US families (25.8%) compared to non-US families (31.9%; χ^2^ = 1.19, *P* = 0.28) (Supplementary Table 6). Inconclusive results were present in 14.4% of URM individuals compared to 9.2% of non-URM individuals (χ^2^ = 1.22, *P* = 0.27), and in 14.9% of US individuals and 8.3% of non-US individuals (χ^2^ = 2.21, *P* = 0.14). We also stratified families by URM and US status together (Supplementary Table 6) and diagnostic yield and rate of inconclusive case classifications did not differ across these joint categories. For the prenatal families, the diagnostic yield was also similar between URM (16.0%) and non-URM (15.4%) families (χ^2^ = 0.13, *P* = 0.72) and did not differ by the number of URM parents (13.0% for two URM versus 22.2% for one URM parent, Table S5); the same was true for US (17.8%) versus non-US families (20.0%) (χ^2^ = 0.27, *P* = 0.60; Table S6). Likewise, the inconclusive rates were 6.7% for URM versus 3.3% for non-URM families (χ^2^ = 1.34, *P* = 0.25) and 7.5% for US versus 5.3% for non-US families (χ^2^ = 0.66, *P* = 0.42). Joint analysis of URM and US status also revealed no significant differences (Supplementary Table 6).

### Parental age effects

Parental age was higher for de novo variants with AD inheritance, with a mean paternal age at conception of 35.3 years for fathers of pediatric patients with de novo variants compared to a mean age of 32.3 years for inherited AD and AR variants (*P* = 0.05) and a mean age of 32.1 years for patients receiving negative results (Supplementary Table 7). Maternal age was also higher, with a mean maternal age of 30.8 years for mothers of pediatric patients with de novo variants with AD inheritance compared to a mean maternal age of 28.4 years for inherited variants (*P* = 0.021) and 28.5 years for patients receiving negative results. For prenatal patients, parental ages were also increased for de novo AD variants, but the increase was not statistically significant (Supplementary Table 7).

### Distribution of variant types

As anticipated, the type of variant (frameshift, stop, missense, in-frame deletion, and splice-site) correlated with the degree of diagnostic certainty. In the entire group, an analysis of the association of variant type with case classification showed that definitive positive patients had the highest frequency of frameshift variants (27.0%), followed by patients with probable positive results (19.5%) and patients with inconclusive results (9.7%, Supplementary Table 8). This pattern was similar for variants predicting stop-gain/loss, with 29.8% in patients with definitive positive results, 14.3% in patients with probable positive results and 7.5% in patients with inconclusive results. In contrast, missense variants were present in 35.5% of patients with definitive positive results, 55.8% of patients with probable positive results, and 72.0% of patients with inconclusive results. In-frame deletions and splice-site variants were infrequent and showed no clear differences among the case classifications.

### Secondary findings

Overall, 712 patients opted to receive secondary findings, including 266/316 prenatal patients (85.0%) and 446/529 pediatric patients (84.3%), as reported previously^23^. There were 26 secondary findings that were reported, 14 in pediatric patients (2.6%) and 12 in prenatal patients (3.8%, χ^2^ = 0.88, *P* = 0.348) (data not shown).

### Multivariate analyses

In a multinomial multivariate analysis of case outcome versus sex, prenatal vs pediatric, URM status, US status, maternal age, paternal age, maternal education, household language, insurance, MUA status, HPSA status, and number of family members sequenced, the beta for a diagnostic outcome in pediatric versus prenatal cases was −0.97 (*P* = 0.0019) for definitive positive, −0.85 (*P* = 0.061) for probable positive, and −0.85 (*P* = 0.035) for inconclusive when compared to negative cases. With a Bonferroni threshold of *P* < 0.0014, paternal age, URM, and US status were not statistically significant, nor was the number of family members sequenced or the remainder of the covariates listed above (Supplementary Table 9).

## Discussion

In this cohort of predominantly US and URM patients that includes both pediatric and prenatal cases, we identified P or LP variants that explained the clinical presentation in 201/845 (23.8%) of patients. The diagnostic yield was higher in pediatric as compared to prenatal cases, although the yield did not differ significantly between the two groups in individuals that underwent ES in the setting of MCAs. Importantly and with implications for clinical care, the diagnostic yield was not significantly different in the offspring of parents who self-reported a URM race/ethnicity compared to those who self-reported non-Hispanic white race/ethnicity. The diagnostic yield likewise did not differ based on the broader category of US status. Similarly, there was no significant increase in inconclusive results between URM and non-URM individuals and between US and non-US individuals in either the pediatric or prenatal study arms. Our results confirm a comparable diagnostic yield based on URM or US status and therefore support application of this technology in patients with referral indications for ES from different population groups. We did identify an increase in the number of inconclusive results in participants from non-white race/ethnicity, although the numbers were not significant. Similar increases in VUSs have been observed by others and hypothesized to be due to reduced representation of individuals with non-European ancestry in genomic databases^24^; these difference may also reflect a lack of data characterizing rare variants, especially missense variants.

Our data add to our understanding of the diagnostic yield of ES in pediatric and prenatal cases with a high proportion of URM/US individuals. Studies of ES in children report diagnostic yields of 30–35% for trio ES^5,25,26^ with lower rates for singleton ES and these results are similar to the 26.7% of positive cases identified in the pediatric patients. Our cohort is unique, in that we included both prenatal and pediatric cases and analyzed all cases with the same ES pipeline, thus enabling a direct comparison between the two groups. Prior studies of prenatal ES have identified a range for diagnostic yield from 8 to 80%, with the two largest cohorts of prenatal cases reporting diagnostic yields of 8.5% and 10%^6,7^. While overall the diagnostic yield of prenatal cases has been reported to be lower than of pediatric patients, a direct comparison of these groups with comparable analysis has not been previously reported. It is of interest that the diagnostic yield in pediatric and prenatal cases enrolled under the diagnostic category of MCAs was comparable, and this suggests that some of the variation in diagnostic yield may reflect differences in clinical indications for ES, rather than stemming from the time of patient ascertainment. Our results also emphasize the higher diagnostic yield previously associated with multiple anomalies compared to many other indications for ES in both prenatal and pediatric individuals^27,28^.

In the pediatric patients, 98/141 (69.5%), of the total positive results were due to variants with AD inheritance versus 25/141 (17.7%) for variants with AR inheritance, a difference that has been identified by others studying patients with DD/ID^29–32^. For the prenatal patients, this difference was still present, but less marked, with 39/60 (65.0%) total positive results due to variants with AD inheritance versus 15/60 (25.0%) for variants with AR inheritance. Similar to our cohort, de novo variants accounted for 80.9% of diagnosed individuals in one study of predominantly non-consanguineous families^31^. Recent studies have also reported on numerous causative genes with AD inheritance associated with MCAs and neurodevelopmental disorders^29^ and a de novo variant with verified paternity and maternity provides strong evidence for pathogenicity according to American College of Medical Genetics and Genomics (ACMG) criteria^25^. Similar to previous studies, paternal age was higher for de novo variants with AD inheritance compared to other inherited variants in our work, consistent with prior evidence indicating that advanced paternal age confers a risk of congenital disorders due to the increased occurrence of de novo variants^33^. Consistent with our results, recent work has also identified an increased risk of de novo variants with increased maternal age, albeit with a lesser effect size when compared to paternal age^34^.

Our pediatric and prenatal cohorts were different with regards to enrollment of URM and US patients, with a greater proportion of URM and US status in the pediatric patients. The high inclusion rates in both patient groups demonstrate the interest that these groups have in genetic testing when this is available. Variation in referral patterns and other recruitment practices between the two study groups, as well as known differences in acceptance of prenatal diagnosis with amniocentesis^35^, may also have contributed to the difference in URM and US recruitment in our two groups. For the pediatric families, the self-reported race/ethnicity distribution in Table 1 closely reflects the distribution of all cases seen in Pediatric Genetics Clinic at UCSF during a similar time period with the exception of a lower proportion of Hispanic/Latino (34.1%) and a higher proportion of white/European (36.5%) in the Pediatric Genetics Clinic (Supplementary Table 10). For the prenatal families, the race/ethnicity distribution was again similar to that observed in the general Ob/Gyn clinics, with the exception of more Black (4.4%) and Pacific Islander (4.9%) and fewer Asian (15.4%) and “multiple/other” (7.9%) patients in the general clinics. The variation in self-identified race/ethnicity between the pediatric and prenatal families likely reflects several factors, including the demographics of patients receiving care at these institutions, pediatric versus maternal fetal medicine clinics, and differences in individuals who requested prenatal or pediatric genetic testing. Prenatal diagnosis has been promoted as an option primarily for patients who might consider pregnancy termination^36^, but with the increased use of ES and detection of disorders for which management options are available, pretest counseling should reflect the possibility that pre- or postnatal interventions may be available to improve outcomes.

In terms of US status, we also compared the geographic distribution of pediatric and prenatal families to the Pediatric Genetics and general Obstetric/Gynecology Clinics, based on zip codes of residence (Supplementary Table 11). First, we note that 2 of 529 (0.4%) pediatric families were from outside of California, versus 2.0% in the Pediatric Genetics Clinics, while 58 of 316 (18.4%) prenatal patients were recruited from 20 states outside of California compared to 2.0% in the general Ob/Gyn clinics (data not shown). For pediatric P^3^EGS patients recruited from California, most came from Northern or Central California and the distribution was quite comparable to the Pediatric Genetics Clinics with the exception of somewhat more participants from the Central valley, including Fresno, San Joaquin and Stanislaus counties (Supplementary Table 11). For the prenatal cases from California, the recruitment pattern was different from the general Ob/Gyn clinics, with relatively more from Contra Costa, Fresno, Santa Clara, Santa Cruz, Stanislaus and Tulare Counties and fewer from Marin, Mendocino, Monterey, and San Francisco counties.

There is also a potential concern that 16.3% of pediatric parents and 19.3% of prenatal parents were missing race/ethnicity and URM information. Although we used structured self-reported race/ethnicity information from a harmonized survey for URM assessment, we also obtained unstructured race/ethnicity information from screening and elegiblity checklist forms (i.e., intake forms) with most prospective parents. In a comparative analysis of intake forms and demographic information obtained by the later survey among 652 families with information from both, 66.0% were URM and 34% were not URM by intake form; for 124 families with intake form data but missing race/ethnicity data from the later demographic survey, 62.9% were URM and 37.1% were not URM by screening form (data not shown). Thus, it appears there was little to no bias in URM status for those missing race/ethnicity information from the survey.

In summary, in this diverse cohort of prenatal and pediatric patients, we identified an overall diagnostic yield of 23.8%. We did not identify differences in diagnostic yield based on non-white race/ethnicity or based on other categories of US status, suggesting that ES has wide utility in these populations. Further investigation of clinical utility in these groups is warranted to determine whether these diagnoses improve outcomes for patients.

## Methods

### Individuals studied and demographics

Patients were enrolled at the UCSF Benioff Children’s Hospital Mission Bay and the Betty Irene Moore Women’s Hospital. Pediatric patients were also enrolled at the Zuckerberg San Francisco General Hospital, UCSF Benioff Children’s Hospital Oakland and the Community Medical Center in Fresno from August 2017 through April 2021. Prenatal patients were also recruited from collaborating groups across the country. Parental race and ethnicity information was obtained by self-report on a harmonized survey. URM pediatric and prenatal cases were defined as having at least one biological parent who self-identified as belonging to any non-white racial or ethnic minority group. If the information on one parent was missing, the child was considered URM if the responding parent was URM; if the responding parent was white or if information was missing for both parents, the self-identified race/ethnicity was considered unknown. Patients were defined as US if they fulfilled one or more of the following three criteria: (1) covered by MediCal health insurance (California’s Medicaid option for low-income families), (2) living in a medically underserved area (MUA), as determined by the home zip code collected from the electronic medical record belonging to the patient and according to the Health Resources and Services Administration (HRSA) shortage designation criteria as listed on their website, and (3) living in a health professional shortage area (HPSA), as determined by the home zip code collected from the electronic medical record belonging to the patient, according to the HRSA shortage designation criteria.

The study was approved by the UCSF Institutional Review Board (IRB) (protocols 17-22504 and 17-22420), the Fresno Community Medical Center IRB (protocol 2019024), and was registered as two clinical trials (“Clinical Utility of Pediatric Whole Exome Sequencing”, NCT03525431 and “Clinical Utility of Prenatal Whole Exome Sequencing”, NCT03482141). Written informed consent was provided by adult participants ≥18 years of age, or by parents or legal guardians on behalf of their children <18 years of age or ≥18 years of age who were unable to consent independently. Assent was obtained from minors and intellectually disabled adults whenever possible. The study was started on 8.1.2017 and completed on 5.13.2022.

### Patient recruitment

We offered testing to patients seen in clinic for whom ES was clinically indicated, with a priority for US and URM families. Eligibility for pediatric and prenatal patients is described in Supplementary Table 12. Pediatric patients were enrolled with the following indications: MCAs, DD/ID, metabolic disease, epilepsy, neurodegenerative disease/cerebral palsy (CP), and encephalopathy. Patients with MCA, metabolic disease, epilepsy, and neurodegenerative disease/CP were further categorized as having, or not having, ID. Prenatal eligibility criteria (Supplementary Table 12) were based on imaging at the time of enrollment, and included one or more fetal structural abnormalities, an unexplained disorder of fetal growth, and one or more fetal effusions or non-immune hydrops. We supported the families with interpreting services and study staff who spoke Spanish. For the pediatric patients, the patient population seen at the Benioff Children’s Hospitals in San Francisco and Oakland was diverse and we did not require specific community outreach efforts for patient recruitment.

We used a modification of the guidelines of Manning et al.^37^ and ordered a microarray for patients with multiple anomalies, DD/ID, and/or autism prior to study entry. We also ordered microarray for growth delays, including short stature, failure to thrive or microcephaly, and neurological findings such as hypotonia and seizures. Patients with a diagnosis that explained their clinical findings after microarray were excluded from the study. We included patients with metabolic diseases because of the high actionability of these conditions. Almost all Pediatric patients were resident in California and were likely to have had non-diagnostic newborn screening prior to enrollment. Lastly, families with children with complex medical conditions may qualify for MediCal and these families were also considered for the study. In the prenatal cohort, we offered enrollment to all patients seen at UCSF with one or more fetal structural anomalies, an unexplained disorder of fetal growth, or one or more fetal effusions. All prenatal cases had to have undergone prenatal diagnosis with nondiagnostic chromosomal microarray. Because many important phenotypic features (e.g., neurologic abnormalities) are not detectable in the fetus, we had a broad inclusion criteria to better understand the prevalence of genetic variants in cases with a single, seemingly isolated anomaly. Indeed the literature supports that most patients with a single anomaly and a genetic variant will have additional ultrasound findings detected later in pregnancy.

### Exome sequencing methodology

Clinical ES was performed at UCSF^38^ in a Clinical Laboratory Improvement Amendments (CLIA) licensed laboratory, the UCSF Clinical Cancer Genomics Laboratory (CLIA number: 05D2034158). Written, informed consent was obtained for study participation. Trio ES including both biological parents was initially undertaken whenever both biological parents were available, while in cases where only one biological parent was available, duo ES was completed. In cases with a prior sibling or fetus affected by a similar phenotype, quad (or greater) ES including the additional affected sibling(s) was performed when possible. Given the urgency of turnaround time for prenatal cases with ongoing pregnancies, a trio approach was undertaken in most cases, while those with a pregnancy termination or loss were sequenced using the ‘proband first’ approach. All patients were provided with the option to receive secondary findings as per ACMG guidelines^39^. In the last year of enrollment, the analysis pipeline was modified to sequence patients with a ‘proband first’ approach to conserve resources, and parents underwent Sanger sequencing only if segregation analysis was required for a reportable variant.

ES analysis was performed as a clinical test using a bioinformatics pipeline developed by the Institute for Human Genetics (IHG) at UCSF. Exon regions were targeted in extracted genomic DNA from probands and biological parents using the xGen Whole Exome Panel kit (Integrated DNA Technologies). Targeted regions were sequenced using the Illumina HiSeq 2500 sequencing system (v3 chemistry) with 100 bp paired-end reads in rapid run mode. The resulting DNA sequences were mapped to and analyzed in comparison with the published human genome (UCSC hg19 reference sequence). The Ingenuity Variant Analysis (IVA, Qiagen) program was used to filter out likely benign variants and to analyze the proband for candidate de novo, homozygous, compound heterozygous and inherited heterozygous variants that were possibly disease causing. Several filters were applied in a stepwise fashion: confidence filter, common variant filter, predicted deleterious filter, custom filters (elimination of common variants ~3 or more alleles from 80 geographically diverse controls- and pseudo-autosomal regions). The UCSF bioinformatics pipeline utilized five different genotype callers for variant calling. To reduce the high number of false positive calls that originate from variants called by a single variant caller, in performing de novo analysis, only variants called by two or more variant callers were analyzed. For inherited heterozygous variants, lower allele frequency cut-off (0.1%) and a patient specific primary gene list were also used for filtering.

Human Gene Mutation Database-Professional (HGMD-Pro), ClinVar and Online Mendelian Inheritance in Man (OMIM) databases were evaluated both for gene-specific variants and gene-disease relationships. Pubmed, Pubmed Central and Google Scholar were also used when no well-defined gene-disease relationship was established in HGMD-Pro and OMIM and if these databases did not include the specific gene variant identified after filtering as described above. Candidate variants were evaluated using the ACMG criteria^24^ and designated as P, LP, or as a VUS^40^. All patients received a case classification at sign-out as either definitive positive, probable positive, inconclusive, or negative. We used a modification of the classification scheme that was developed by the Sequencing and Diagnostic Yield (SADY) Working Group within the Clinical Sequencing Evidence-Generating Research (CSER) consortium (Supplementary Table 13)^26,41^. Variants in genes with clinical overlap with the patient’s phenotype were reported to study participants. Only one proband was counted for each family in which more than one individual was affected. Reanalysis was performed in some cases, but only the initial ES results are included in this report. Mitochondrial genome variants were unable to be detected with our analysis pipeline.

Secondary findings were only assessed in the proband initially in this study, so the proband first approach did not influence availability of secondary findings. We confirmed all pathogenic/likely pathogenic variants by Sanger sequencing in proband and parents, a decision made by the clinical laboratory. In some situations, segregation was determined for a VUS in a gene with a strong gene–disease relationship, or an emerging gene–disease relationship, to determine if the VUS met reportability criteria. We adhered to a high standard for declaring variants as P or LP and often designated variants as VUSs due to the limitations of ACMG classification algorithm, even if the VUSs were considered likely to explain the patient phenotype by the referring clinician.

### Statistical methods

Basic univariate analyses of discrete outcomes were performed using chi-squared tests, with two-tailed *P*-values of 0.05 for nominal statistical significance. For continuous parametric variables, t-tests were performed with similar *P*-values. A Bonferroni correction was applied to the threshold of significance to correct for multiple comparisons. For multivariable analyses, we used R version 4.0.5 for statistical calculations. In addition to the built-ins, we utilized the mgcv library for generalized additive model regression and the ordinal library for ordinal regression. We examined diagnostic yield (definitive positive and probable positive case classifications) based on sex, the ages of pediatric patients, the ages of parents at the time of conception and at the time of enrollment for pediatric patients, URM status, US status, indications for genetic testing and diagnostic categories, and exome approach (proband first, duo, trio or quad). Mode of inheritance of the causative gene(s), comprising AD, AR, or XL, was also analyzed. We compared diagnostic yield between prenatal and pediatric patients, adjusting for confounders and differences between groups. Finally, we created a multinomial regression model to evaluate variables influencing both diagnostic yield and inconclusive rates; this model included exome approach, parental age at conception, URM and US status, and clinical indications for testing within and between the pediatric and prenatal patients. This regression allowed for calculation of odds ratios for the case classifications of definite positive, probable positive, and inconclusive relative to a negative outcome.

### Reporting summary

Further information on research design is available in the Nature Research Reporting Summary linked to this article.

### Supplementary information

Supplementary Tables

Reporting Summary

## Data Availability

Sequencing data has been uploaded to the Analysis Visualization and Informatics Lab-space (AnVIL) at the National Human Genome Research Institute. Clinical data is available from the authors on reasonable request. The sequencing was performed in a in a Clinical Laboratory Improvement Amendments (CLIA) licensed laboratory, the UCSF Clinical Cancer Genomics Laboratory (CLIA number is: 05D2034158). A list of pathogenic or likely pathogenic primary variants is provided in Supplementary Table 14.

## Code availability

No custom code or scripts were used.
