# Author Correction: Diagnostic yield of pediatric and prenatal exome sequencing in a diverse population

**DOI:** 10.1038/s41525-023-00382-9

**Published:** 2023-10-23

**Authors:** Anne Slavotinek, Shannon Rego, Nuriye Sahin-Hodoglugil, Mark Kvale, Billie Lianoglou, Tiffany Yip, Hannah Hoban, Simon Outram, Beatrice Anguiano, Flavia Chen, Jeremy Michelson, Roberta M. Cilio, Cynthia Curry, Renata C. Gallagher, Marisa Gardner, Rachel Kuperman, Bryce Mendelsohn, Elliott Sherr, Joseph Shieh, Jonathan Strober, Allison Tam, Jessica Tenney, William Weiss, Amy Whittle, Garrett Chin, Amanda Faubel, Hannah Prasad, Yusuph Mavura, Jessica Van Ziffle, W. Patrick Devine, Ugur Hodoglugil, Pierre-Marie Martin, Teresa N. Sparks, Barbara Koenig, Sara Ackerman, Neil Risch, Pui-Yan Kwok, Mary E. Norton

**Affiliations:** 1 Department of Pediatrics, University of California, San Francisco, San Francisco, CA USA; 2 Institute for Human Genetics, University of California San Francisco, San Francisco, CA USA; 3 Department of Epidemiology & Biostatistics, University of California San Francisco, San Francisco, CA USA; 4 Department of Surgery, University of California, San Francisco, San Francisco, CA USA; 5 Institute for Health & Aging, School of Nursing, University of California San Francisco, San Francisco, CA USA; 6 Institute of Human Nutrition, Columbia University Medical Center, New York, NY USA; 7 Division of Pediatric Neurology, Department of Pediatrics, University of Louvain, Brussels, Belgium; 8 Genetic Medicine, University of California, San Francisco, Fresno, CA USA; 9 Department of Neurology, UCSF Benioff Children’s Hospital Oakland, Oakland, CA USA; 10 Eysz, Inc, Piedmont, CA USA; 11 Division of Genetics, Kaiser Permanente Oakland Medical Center, Oakland, CA USA; 12 Division of Child Neurology, Department of Neurology, University of California, San Francisco, San Francisco, CA USA; 13 Division of Child Neurology, Zuckerberg San Francisco General Hospital, San Francisco, San Francisco, CA USA; 14 Division of Pediatrics, Zuckerberg San Francisco General Hospital, San Francisco, San Francisco, CA USA; 15 Department of Pathology, University of California San Francisco, San Francisco, CA USA; 16 Genomic Medicine Laboratory, University of California San Francisco, San Francisco, CA USA; 17 Division of Maternal Fetal Medicine, Department of Obstetrics, Gynecology, and Reproductive Sciences, San Francisco, USA; 18 Program in Bioethics, University of California, San Francisco, San Francisco, CA USA; 19 Department of Social & Behavioral Sciences, School of Nursing, University of California San Francisco, San Francisco, CA USA; 20 Cardiovascular Research Institute, University of California San Francisco, San Francisco, CA USA

**Keywords:** Neurological disorders, Molecular medicine, Genetic testing

Correction to: *npj Genomic Medicine* 10.1038/s41525-023-00353-0, published online 26 May 2023

The wrong Supplementary file was originally published with this article; it has now been replaced with the correct version. The original article has been corrected.

### Supplementary information

Supplementary Information

