# Supplementary material for: Diagnostic yield of pediatric and prenatal exome sequencing in a diverse population
Source: NPJ Genom Med. 2023 May 26;8:10. doi: 10.1038/s41525-023-00353-0 (PMC10220040; doi:10.1038/s41525-023-00353-0)
Supplement: Supplementary file 1 — Supplementary Tables [file 41525_2023_353_MOESM1_ESM.pdf]

Supplementary table 1. Age at Enrollment, Diagnostic Yield and Inconclusive Rate for Pediatric Patients

**Proband age at enrollment**	n	Positive	Inconclusive	Negative
Newborn to up to 1 year old	86 (.163)	24 (.279)	15 (.174)	47 (.547)
1-2 years old	97 (.183)	25 (.258)	12 (.126)	59 (.621)
3-5 years old	112 (.212)	26 (.232)	14 (.125)	72 (.643)
6-10 years old	110 (.208)	32 (.291)	16 (.145)	62 (.564)
11-15 years old	93 (.176)	24 (.258)	10 (.108)	59 (.634)
16-25 years old	31 (.059)	10 (.323)	7 (.226)	14 (.452)
**Total**	**529 (1.000)**			

Supplementary table 2. Pregnancy Outcomes, Diagnostic Yield and Inconclusive Rate for Prenatal Patients

**Pregnancy outcome**	n	Positive	Inconclusive	Negative
Stillbirth (>20 weeks)	19 (.064)	2 (.105)	1 (.053)	16 (.842)
Living child	122 (.380)	10 (.082)	10 (.082)	102 (.936)
Neonatal/living child death	33 (.105)	9 (.273)	3 (.091)	21 (.636)
Pregnancy termination	135 (.427)	37 (.274)	5 (.037)	93 (.689)
Miscarriage (<20 weeks)	7 (.022)	2 (.286)	1 (.143)	4 (.571)
**Total**	**316 (1.000)**			

Supplementary table 3. Diagnostic Yield (Proportion Positive) and Inconclusive Rate by Mode of Inheritance, Sequencing Approach and Sex of Proband for Pediatric Patients

Result type		Quad (n = 19)	Trio (n = 326)	Duo (n = 108)	Proband first (n = 76)	Female (n = 239)	Male (n = 290)	Total (n = 529)
**Definitive positive**	**Mode of inheritance**							
	AD[1], *de novo*	1 (.053)	46 (.141)	1 (.009)	5 (.066)	25 (.105)	28 (.097)	53 (.100)
	AD, inherited	3 (.157)	5 (.015)	0 (.000)	0 (.000)	4 (.017)	4 (.014)	8 (.015)
	AD, segregation unknown	0 (.000)	0 (.000)	6 (.056)	2 (.026)	2 (.008)	6 (.021)	8 (.015)
	AR[2], HZ[3]	1 (.053)	6 (.018)	2 (.019)	1 (.013)	7 (.029)	3 (.010)	10 (.019)
	AR, comp. het.[4]	0 (.000)	4 (.012)	0 (.000)	1 (.013)	2 (.008)	3 (.010)	5 (.009)
	X-linked	0 (.000)	11 (.034)	1 (.009)	0 (.000)	10 (.042)	2 (.007)	12 (.022)
	All	5 (.263)	72 (.221)	10 (.093)	9 (.118)	50 (.209)	46 (.159)	96 (.181)
**Probable positive**	**Mode of inheritance**							
	AD *de novo*	0 (.000)	11 (.034)	0 (.000)	3 (.039)	10 (.042)	4 (.014)	14 (.026)
	AD inherited	0 (.000)	3 (.009)	2 (.019)	1 (.013)	3 (.013)	3 (.010)	6 (.011)
	AD unknown	0 (.000)	0 (.000)	8 (.074)	1 (.013)	3 (.013)	6 (.021)	9 (.017)
	AR HZ	0 (.000)	3 (.009)	1 (.009)	0 (.000)	3 (.013)	1 (.003)	4 (.008)
	AR comp het	0 (.000)	2 (.006)	4 (.037)	0 (.000)	5 (.021)	1 (.003)	6 (.011)
	X-linked	0 (.000)	3 (.009)	2 (.019)	1 (.013)	3 (.013)	3 (.010)	6 (.011)
	All	0 (.000)	22 (.067)	17 (.157)	6 (.079)	27 (.113)	18 (.062)	45 (.085)
**Definitive/probable positive**	**Mode of inheritance**							
	AD *de novo*	1 (.053)	57 (.175)	1 (.009)	8 (.105)	35 (.146)	32 (.110)	67 (.127)
	AD inherited	3 (.157)	8 (.025)	2 (.019)	1 (.013)	7 (.029)	7 (.024)	14 (.026)
	AD unknown	0 (.000)	0 (.000)	14 (.13)	3 (.039)	5 (.021)	12 (.041)	17 (.032)
	AR HZ	1 (.053)	9 (.028)	3 (.028)	1 (.013)	10 (.042)	4 (.014)	14 (.026)
	AR comp het	0 (.000)	6 (.018)	4 (.037)	1 (.013)	7 (.029)	4 (.014)	11 (.021)
	X-linked	0 (.000)	14 (.043)	3 (.028)	1 (.013)	13 (.054)	5 (.017)	18 (.034)
	All	5 (.263)	94 (.288)	27 (.25)	15 (.197)	77 (.322)	64 (.221)	141 (.267)
**Inconclusive**	**Mode of inheritance**							
	AD *de novo*	0 (.000)	10 (.031)	0 (.000)	1(.013)	6 (.025)	5 (.017)	11 (.021)
	AD inherited	0 (.000)	7 (.021)	2 (.019)	2 (.026)	4 (.017)	7 (.024)	11 (.021)
	AD unknown	0 (.000)	1 (.003)	7 (.065)	0 (.000)	4 (.017)	4 (.014)	8 (.015)
	AR HZ	1 (.053)	17 (.052)	5 (.046)	1 (.013)	9 (.038)	15 (.052)	24 (.045)
	AR comp het	0 (.000)	5 (.015)	4 (.037)	0 (.000)	5 (.021)	4 (.014)	9 (.017)
	X-linked	0 (.000)	9 (.028)	2 (.019)	0 (.000)	4 (.017)	7 (.024)	11 (.021)
	All	1 (.053)	49 (.150)	20 (.185)	4 (.053)	32 (.134)	42 (.145)	74 (.140)
**Negative**		13 (.684)	183 (.561)	61 (.565)	57 (.75)	130 (.544)	184 (.634)	314 (0.594)

AD[1] = autosomal dominant; AR[2] =- autosomal recessive; HZ[3] = homozygous; comp. het.[4] = compound heterozygous.

Supplementary table 4. Diagnostic Yield (Proportion Positive) and Inconclusive Rate by Mode of Inheritance, Sequencing Approach and Sex of Proband for Prenatal Patients

		Quad* (n = 12)	Trio (n = 257)	Duo (n = 14)	Proband first (n = 33)	Female (n = 145)	Male (n = 171)	Total (n = 316)
**Definitive positive**	**Mode of inheritance**							
	AD[1], *de novo*	0 (.000)	26 (.101)	1 (.071)	1 (.030)	11 (.076)	17 (.099)	28 (.089)
	AD, inherited	0 (.000)	0 (.000)	0 (.000)	1 (.030)	1 (.007)	0 (.000)	1 (.003)
	AD, segregation unknown	0 (.000)	0 (.000)	1 (.071)	0 (.000)	0 (.000)	1 (.006)	1 (.003)
	AR[2], HZ[3]	0 (.000)	2 (.008)	0 (.000)	0 (.000)	1 (.007)	1 (.006)	2 (.006)
	AR, comp. het.[4]	0 (.000)	1(.004)	0 (.000)	3 (.091)	2 (.014)	2 (.012)	4 (.013)
	X-linked	0 (.000)	3 (.012)	0 (.000)	0 (.000)	2 (.014)	1 (.006)	3 (.009)
	All	0 (.000)	32 (.125)	2 (.143)	5 (.152)	17 (.117)	22 (.129)	39 (.123)
**Probable positive**	**Mode of inheritance**							
	AD *de novo*	0 (.000)	6 (.023)	0 (.000)	0 (.000)	3 (.021)	3 (.018)	6 (.019)
	AD inherited	0 (.000)	3 (.012)	0 (.000)	0 (.000)	1 (.007)	2 (.012)	3 (.009)
	AD unknown	0 (.000)	0 (.000)	0 (.000)	0 (.000)	0 (.000)	0 (.000)	0 (.000)
	AR HZ	2 (.167)	0 (.000)	0 (.000)	0 (.000)	0 (.000)	2 (.012)	2 (.006)
	AR comp het	0 (.000)	4 (.016)	0 (.000)	3 (.091)	5 (.034)	2 (.012)	7 (.022)
	X-linked	0 (.000)	3 (.012)	0 (.000)	0 (.000)	0 (.000)	3 (.018)	3 (.009)
	All	2 (.167)	16 (.062)	0 (.000)	3 (.091)	9 (.062)	12 (.070)	21 (.066)
**Definitive/probable positive**	**Mode of inheritance**							
	AD *de novo*	0 (.000)	32 (.125)	1 (.071)	1 (.030)	14 (.097)	20 (.117)	34 (.108)
	AD inherited	0 (.000)	3 (.012)	0 (.000)	1 (.030)	2 (.014)	2 (.012)	4 (.013)
	AD unknown	0 (.000)	0 (.000)	1 (.071)	0 (.000)	0 (.000)	1 (.006)	1 (.003)
	AR HZ	2 (.167)	2 (.008)	0 (.000)	0 (.000)	1 (.007)	3 (.018)	4 (.013)
	AR comp het	0 (.000)	5 (.019)	0 (.000)	6 (.182)	7 (.048)	4 (.023)	11 (.035)
	X-linked	0 (.000)	6 (.023)	0 (.000)	0 (.000)	2 (.014)	4 (.023)	6 (.019)
	All	2 (.167)	48 (.187)	2 (.143)	8 (.242)	26 (.179)	34 (.199)	60 (.190)
**Inconclusive**	**Mode of inheritance**							
	AD *de novo*	1 (.083)	5 (.019)	0 (.000)	0 (.000)	5 (.034)	1 (.006)	6 (.019)
	AD inherited	0 (.000)	4 (.016)	0 (.000)	0 (.000)	0 (.000)	4 (.023)	4 (.013)
	AD unknown	0 (.000)	1 (.004)	1 (.071)	0 (.000)	1 (.007)	1 (.006)	2 (.006)
	AR HZ	0 (.000)	2 (.008)	0 (.000)	1 (.030)	2 (.014)	1 (.006)	3 (.009)
	AR comp het	0 (.000)	2 (.008)	0 (.000)	1 (.030)	0 (.000)	3 (.018)	3 (.009)
	X-linked	0 (.000)	2 (.008)	0 (.000)	0 (.000)	0 (.000)	2 (.012)	2 (.006)
	All	1 (.083)	16 (.062)	1 (.071)	2 (.061)	8 (.055)	12 (.070)	20 (.063)
**Negative**		9 (.750)	193 (.751)	11 (.786)	23 (.697)	111 (.765)	125 (.730)	236 (.747)

*One quintet included in Quads. AD[1] = autosomal dominant; AR[2] =- autosomal recessive; HZ[3] = homozygous; comp. het.[4] = compound heterozygous.

Supplementary table 5. Diagnostic Yield (Proportion Positive) and Inconclusive Rate by Number of Underrepresented Minority (URM) Parents for Pediatric and Prenatal Patients

	2 URM	1 URM, 1 Not	1 URM, 1 ?	≥1 URM	2 Not	1 Not, 1?	2 ?
**Pediatric**							
Definitive Positive	51 (.171)	11 (.196)	6 (.162)	68 (.173)	12 (.185)	1 (.111)	15 (.238)
Probable Positive	24 (.080)	5 (.089)	5 (.135)	34 (.087)	6 (.092)	0 (.000)	5 (.079)
All Positive	75 (.251)	16 (.286)	11 (.297)	102 (.260)	18 (.277)	1 (.111)	20 (.317)
Inconclusive	43 (.144)	10 (.179)	4 (.108)	57 (.145)	6 (.092)	2 (.222)	9 (.143)
Negative	181 (.605)	30 (.536)	22 (.595)	233 (.594)	41 (.631)	6 (.667)	34 (.540)
Total	299	56	37	392	65	9	63
**Prenatal**							
Definitive Positive	9 (.083)	6 (.133)	2 (.200)	17 (.104)	9 (.099)	3 (.250)	10 (.200)
Probable Positive	5 (.046)	4 (.089)	0 (.000)	9 (.055)	5 (.055)	2 (.167)	5 (.100)
All Positive	14 (.130)	10 (.222)	2 (.200)	26 (.160)	14 (.154)	5 (.417)	15 (.300)
Inconclusive	10 (.093)	1 (.022)	0 (.000)	11 (.067)	3 (.033)	2 (.167)	4 (.080)
Negative	84 (.778)	34 (.756)	8 (.800)	126 (.773)	74 (.813)	5 (.417)	31 (.620)
Total	108	45	10	163	91	12	50

URM=underrepresented minority parent; Not=white parent; ? = race/ethnicity missing

Supplementary table 6. Diagnostic Yield (Proportion Positive) and Inconclusive Rate by Underserved and Underrepresented Minority Status for Pediatric and Prenatal Patients

	US	Not US	US, URM	US, Not URM	US, URM?	Not US, URM	Not US, Not URM	Not US, URM?
**Pediatric**								
Definitive Positive	80 (.175)	16 (.222)	61 (.167)	8 (.216)	11 (.200)	7 (.219)	5 (.172)	4 (.364)
Probable Positive	38 (.083)	7 (.097)	31 (.085)	1 (.027)	6 (.109)	2 (.063)	5 (.172)	0 (.000)
All Positive	118 (.258)	23 (.319)	92 (.252)	9 (.243)	17 (.309)	9 (.281)	10 (.345)	4 (.364)
Inconclusive	68 (.149)	6 (.083)	56 (.153)	4 (.108)	8 (.145)	4 (.125)	1 (.034)	1 (.091)
Negative	271 (.593)	43 (.597)	217 (.595)	24 (.6490	30 (.545)	19 (.594)	18 (.621)	6 (.545)
Total	457	72	365	37	55	32	29	11
**Prenatal**								
Definitive Positive	20 (.137)	19 (.112)	8 (.100)	5 (.167)	7 (.194)	10 (.128)	5 (.086)	4 (.118)
Probable Positive	6 (.041)	15 (.088)	4 (.050)	1 (.033)	1 (.028)	5 (.064)	3 (.052)	7 (.206)
All Positive	26 (.178)	34 (.200)	12 (.150)	6 (.200)	8 (.222)	15 (.192)	8 (.138)	11 (.324)
Inconclusive	11 (.075)	9 (.053)	6 (.075)	1 (.033)	4 (.111)	6 (.077)	2 (.034)	1 (.029)
Negative	109 (.747)	127 (.747)	62(.775)	23 (.767)	24 (.667)	57 (.731)	48 (.828)	22 (.647)
Total	146	170	80	30	36	78	58	34

Supplementary table 7. Parental Age at Conception According to Variant Type and Inheritance (years, with fraction of years)

**Variant type**	**Pediatric**		**Prenatal**	
-	**Mother**	**Father**	**Mother**	**Father**
**Autosomal dominant, *de novo***	30.8	35.3	33.2	35.3
**Other inherited variants, excluding X-linked variants**	28.4	32.3	30.4	32.1
**Negative**	28.5	32.1	32.8	35.20

Supplementary table 8. Variant Type in Patients with Definitive Positive, Probable Positive and Inconclusive Results for both Pediatric and Prenatal Patients

	**Definitive positive**	**%**	**Probable positive**	**%**	**Inconclusive**	**%**
Frameshift	38	27.0	15	19.5	9	9.7
Stop-gain/loss	42	29.8	11	14.3	7	7.5
Missense	50	35.5	43	55.8	67	72.0
In-frame deletion	2	1.4	1	1.3	3	3.2
Splice-site	9	6.4	7	9.1	7	7.5
Total	141	100.0	77	100.0	93	100.0

Supplementary table 9. Results of Multinomial Regression Analysis

A. Definitive Positive vs Negative

	Beta	Standard error	P-value
(Intercept)	-2.6	0.86	0.0026
Self-identified URM = True	-0.029	0.33	0.93
Self-identified URM = Unknown	-0.063	0.78	0.94
Prenatal case = True	-0.97	0.31	0.0019
Sex = Male	0.33	0.24	0.16
Samples run = 1 vs. 3	-0.51	0.45	0.26
Samples run = 2 vs. 3	-0.015	0.61	0.98
Samples run = 4 vs. 3	-0.47	0.58	0.42
Maternal age (years)	-0.0099	0.028	0.73
Paternal age (years)	0.053	0.021	0.011
Maternal education = 2	-0.36	0.35	0.3
Maternal education = 3	-0.12	0.38	0.75
Household language = Other	-0.28	0.35	0.43
Household language = English or Other	-0.37	0.37	0.31
Insurance = Public	0.24	0.35	0.5
MUA = True	0.64	0.27	0.019
HPSA = True	-0.62	0.36	0.083
Underserved resident = True	0.45	0.45	0.31

B. Probable Positive vs Negative

	Beta	Standard error	P-value
(Intercept)	-2.9	1.3	0.022
Self-identified URM = True	-0.21	0.49	0.67
Self-identified URM = Unknown	1.7	0.77	0.027
Prenatal case = True	-0.85	0.45	0.061
Sex = Male	0.49	0.34	0.15
Samples run = 1 vs. 3	0.019	0.55	0.97
Samples run = 2 vs. 3	1	0.64	0.1
Samples run = 4 vs. 3	-0.6	1.1	0.57
Maternal age (years)	0.034	0.043	0.43
Paternal age (years)	-0.0065	0.036	0.86
Maternal education = 2	0.24	0.52	0.65
Maternal education = 3	-0.06	0.54	0.91
Household language = Other	0.75	0.51	0.14
Household language = English or Other	-0.065	0.57	0.91
Insurance = Public	-0.15	0.52	0.77
MUAP = True	-0.77	0.53	0.15
HPSA = True	-0.55	0.55	0.36
Underserved resident = True	-1	1.1	0.35

C. Inconclusive vs Negative

	Beta	Standard error	P-value
(Intercept)	-0.75	1.1	0.49
Self-identified URM = True	0.82	0.54	0.12
Self-identified URM = Unknown	1.0	1.2	0.4
Prenatal case = True	-0.85	0.4	0.035
Sex = Male	0.28	0.29	0.33
Samples run = 1 vs. 3	-1.2	0.76	0.11
Samples run = 2 vs. 3	0.8	0.52	0.13
Samples run = 4 vs. 3	-0.49	0.78	0.53
Maternal age (years)	-0.036	0.037	0.34
Paternal age (years)	-0.014	0.031	0.65
Maternal education = high scool	-0.19	0.38	0.61
Maternal education = college	-0.15	0.43	0.74
Household language = Other	-0.86	0.43	0.046
Household language = English or Other	0.34	0.36	0.34
Insurance = Public	0.38	0.46	0.4
MUAP = True	-0.0063	0.36	0.99
HPSA = True	-0.37	0.4	0.35
Underserved resident = True	-0.82	0.7	0.24

Supplementary table 10. Race/Ethnicity Distribution of P^{3}EGS Cases Versus UCSF Pediatric Genetics Clinics and Obstetrics/Gynecology Clinics*

	Pediatric		Prenatal	
	**P^{3}EGS**	**Clinics**	**P^{3}EGS**	**Clinics**
Hispanic	.48	.34	.20	.20
Asian	.12	.11	.19	.15
Black/African America	.05	.05	.01	.04
Native American	.01	.01	<.01	<.01
Pacific Islander	.01	.01	<.01	.05
White	.22	.37	.47	.47
Multiple/Other	.10	.11	.13	.08

*Excluding those with missing race/ethnicity information; averaging P^{3}EGS parents

Supplementary table 11. Geographic Distribution (by California County) of P^3EGS Cases Versus UCSF Pediatric Genetics Clinics and Obstetrics/Gynecology Clinics for California Cases

	Pediatric		Prenatal	
	P^3EGS	Clinics	P^3EGS	Clinics
Alameda	.08	.07	.04	.04
Contra Costa	.24	.23	.19	.11
Fresno	.06	.02	.07	<.01
Marin	.07	.08	.05	.16
Mendocino	.09	.09	.03	.11
Monterey	.02	.02	.02	.09
Sacramento	.01	<.01	.02	<.01
San Francisco	.14	.16	.18	.22
San Joaquin	.08	.05	.02	<.01
San Mateo	.03	.05	.09	.10
Santa Clara	<.01	.01	.06	.01
Santa Cruz	<.01	.01	.03	.01
Stanislaus	.08	.06	.06	.01
Tulare	.02	.01	.03	<.01
Other	.07	.14	.11	.13

Supplementary Table 12. Enrollment Criteria for Program in Prenatal and Pediatric Genomic Sequencing (P^3EGS)

**Inclusion Criteria**<br>1. Presenting clinical features suggestive of a genetic etiology, including ID[a], seizures, multiple congenital anomalies, metabolic conditions, and neurodegenerative conditions or idiopathic CP[b]; up to 80 of these patients will have encephalopathy or multiple congenital anomalies so that they may benefit from rapid exome sequencing in the Pediatric Intensive Care Unit or Neonatal Intensive Care Unit.<br>2. Pregnant women with fetuses with structural birth defects identified by ultrasound.<br>3. A minimum of one biological parent is available and willing to provide a biospecimen for ES[c], with a preference for two available parents. At least one parent consenting to ES of the child. For the prenatal cases, at least the mother had to consent to ES of a fetal sample as well as on herself.<br>4. Pediatric patients must have had at least one prior genetics appointment or evaluation.<br>5. All pediatric patients with a clinical indication for chromosomal microarray analysis (CMA) and all prenatal patients were required to have non-diagnostic CMA results prior to enrollment. Pregnancies and patients with a copy number variant not clearly associated with the phenotype were eligible for inclusion, as were patients who had previously undergone targeted or gene panel testing without a diagnosis.<br>6. Pregnant patients late in gestation, in whom ES results were not anticipated until after delivery, were included in the prenatal subgroup if consent occurred prior to delivery.<br>7. Twin gestations were eligible for inclusion if one or both fetuses were affected.
**Exclusion Criteria**<br>1. Prior ES performed for a clinical or research indication<br>2. Lack of phenotypic indication of a likely underlying genetic etiology<br>3. Both biological parents are unavailable

ID[a] = Intellectual disability; CP[b] = Cerebral palsy; ES[c] = exome sequencing. [3]Other includes: Pulmonary hypertension with alveolar capillary dysplasia with misalignment of the pulmonary veins; Apnea and hypoventilation; hypotonia; Immunodeficiency with hypogammaglobulinemia; Crohn's disease; juvenile rheumatoid arthritis; Retinal dystrophy; family history of consanguinity and affected sibling; Skin laxity; Respiratory distress; immunodeficiency; Pituitary mass; Dilated cardiomyopathy; family history of consanguinity; Liver hemangiomas; focal nodular hyperplasia of the liver; recurrent epistaxis; Growth delays; Failure to thrive and short stature; Growth delays.

Supplementary table 13. Definition of Definitive Positive and Probable Positive Case Results for the CSER consortium

Definitive Positive
• Implicated variant(s) are pathogenic<br>• Phenotype and inheritance pattern consistent with condition<br>• Known phase or *de novo* status
Probable Positive
• Implicated variant(s) are likely pathogenic or a combination of pathogenic/likely pathogenic<br>• For recessive condition, combination of a pathogenic/likely pathogenic variant with a variant of uncertain significance provided no other inconclusive conditions (below) exist<br>• Phenotype and inheritance pattern are consistent with condition<br>• Known phase or de novo status, or in recessive condition with only one parent/sibling available, that family member has only one of the implicated variants
Inconclusive
Presence of one or more contributions to case-level ambiguity<br>• Unknown phase<br>• Variant uncertainty<br>• Insufficient zygosity<br>• Phenotype mismatch<br>• Novel gene
Negative
• All other cases

Supplementary table 14

gene	amino acid	transcript	nucleotide change	chromosc	position	end_coordinate	reference	alternate	moi	allele_origin	acmg_classif	zygosity
**genome_build is GRCh37 for all patients**												
GLI2	p.W1146*	NM_005270.4	c.3438G>A	2	121746928	121746928	G	A	AD	De novo	P	het.
MECP2	p.Gln406Ter	NM_004992.3	c.1216C>T	X	153296063	153296063	G	A	XL	De novo	P	het.
KAT6A	p.V1347fs*6	NM_006766.4	c.4038delT	8	41791700	41791700		delA	AD	Unk., father unavailable	P	het.
ARID1A	p.P1898fs*25	NM_006015.5	c.5693delC	1	27106082	27106082		delC	AD	De novo	P	het.
ZIC3	p.R320*	NM_003413.3	c.958C>T	X	136649808	136649808	C	T	XL	Maternal	LP	hemiz.
TANGO2	p.R86*	NM_152906.5	c.256C>T	22	20040098	20040098	C	T	AR	Mat. and Pat. (HZ)	P	HZ
KMT2A	S191*	NM_005933.3	c.572C>A	11	118342446	118342446	C	A	AD	Unk., father unavailable	P	het.
EP300	p.R1055*	NM_001429.3	c.3163C>T	22	41551019	41551019	C	T	AD	De novo	P	het.
DDX3X	p.Arg326Cys	NM_001356.4	c.976C>T	X	41203603	41203603	C	T	XL	De novo	P	het.
TRAF7	p.R655Q	NM_032271.2	c.1964G>A	16	2226351	2226351	G	A	AD	De novo	LP	het.
KAT6A	p.K1410fs*7	NM_006766.4	c.4228_4232delAAAGA	8	41791506	41791510		delTCTTT	AD	Unk., father unavailable	P	het.
GLI2	p.K647fs*48	NM_005270.4	c.1940delA	2	121742303	121742303		delA	AD	Maternal	LP	het.
SRD5A2	p.Tyr235Phe	NM_000348.3	c.704A>T	2	31751326	31751326	T	A	AR	Mat. and Pat. (HZ)	P	HZ
PDHA1	p.T354P	NM_000284.3	c.1060A>C	X	19377658	19377658	A	C	XL	Unk., mother unavailable	LP	het.
EXT2	p.Q424Q	NM_000401.3	c.1272G>A	11	44151688	44151688	G	A	AR	Mat. and Pat. (HZ)	LP	HZ
PDCD10	p.K111fs*15	NM_007217.4	c.333delA	3	167413446	167413446		delT	AD	De novo	P	het.
EP300	p.Y1162*	NM_001429.3	c.3485dupA	22	41553396	41553396	dupA		AD	De novo	P	het.
HNRNPH2	p.R206Q	NM_001032393.2	c.617G>A	X	100667593	100667593	G	A	XL	De novo	P	het.
ANKRD17	p.Ala1920Serfs*20	NM_032217.3	c.5756dupG	4	73957589	73957589	dupG		AD	De novo	LP	het.
BRAF	p.D638E	NM_004333.5	c.1914T>G	7	140449165	140449165	A	C	AD	De novo	P	het.
NALCN	p. Leu1150Ile	NM_052867.3	c.3448C>A	13	101742055	101742055	G	T	AD	De novo	P	het.
KCNA2	G197fs*4	NM_004974.3	c.590delG	1	111146815	111146815		delC	AD	Maternal	P	het.
PRRT2	p.R217fs*8	NM_001256442.1	c.649dupC	16	29825024	29825024	dupC		AD	Maternal	P	het.
KMT2D	p.M999*	NM_003482.3	c.2994delT	12	49444377	49444377		delA	AD	De novo	P	het.
INTS1	p.M1013fs*55	NM_001080453.2	c.3036delC	7	1525046	1525046		delG	AR	Maternal	LP	cpd het.
INTS1		NM_001080453.2	c.3430-2A>C	7	1523491	1523491	T	G	AR	Unk., father unavailable	LP	cpd het.
KMT2A	p.I312fs*10	NM_005933.3	c.934_935insC	11	118342808	118342809	insC		AD	De novo	P	het.
KMT2A	p.Ser2805fs*22	NM_005933.3	c.8405_8409delGCTCA	11	118375021	118375025		delGCTCA	AD	De novo	P	het.
CHD7		NM_017780.3	c.4533+1G>A	8	61750815	61750815	G	A	AD	De novo	P	het.
KRIT1	p.S301*	NM_194456.1	c.902C>G	7	91863850	91863850	G	C	AD	De novo	P	het.
KMT2A	p.R1154W	NM_005933.3	c.3460C>T	11	118348807	118348807	C	T	AD	De novo	P	het.
SOX10	p.S135N	NM_006941.3	c.404G>A	22	38379388	38379388	C	T	AD	De novo	LP	het.
POGZ	p.Q1005fs*5	NM_015100.3	c.3041delA	1	151378470	151378470		delT	AD	Paternal	P	het.
PDHA1	p.R302C	NM_000284.3	c.904C>T	X	19377038	19377038	C	T	XL	De novo	P	het.
TFAP2A	p.R254W	NM_003220.2	c.760C>T	6	10404745	10404745	G	A	AD	De novo	P	het.
FOXF1	p.R138P	NM_001451.2	c.413G>C	16	86544588	86544588	G	C	AD	De novo	LP	het.
SETBP1	p.Ile871Thr	NM_015559.3	c.2612T>C	18	42531917	42531917	T	C	AD	De novo	P	het.
GNAS	p.R231C	NM_000516	c.691C>T	20	57484607	57484607	C	T	AD	De novo	P	het.
SLC26A2	p.P100fs*5	NM_000112.3	c.299delC	5	149357514	149357514		delC	AR	Mat. and Pat. (HZ)	P	HZ
MECP2	p.T158M	NM_004992.3	c.194C>T	X	153297841	153297841	G	A	XL	De novo	P	het.
DGAT1	p.G2fs*65	NM_012079.5	c.5delG	8	145550295	145550295		delC	AR	Mat. and Pat. (HZ)	P	HZ
RAD21	p.R478*	NM_006265.2	c.1432C>T	8	117864225	117864225	G	A	AD	Maternal	P	het.
TCF12	p.R7fs*5	NM_207036	c.19delC	15	57212130	57212130		delC	AD	Unk., father unavailable	LP	het.
PTEN	p.Leu345fs*16	NM_000314.6	c.1032dupG	10	89725049	89725049	dupG		AD	De novo	P	het.
SCN2A	p.K905E	NM_021007.2	c.2317A>G	2	166188007	166188007	A	G	AD	De novo	P	het.
TUBB2A	p.P358T	NM_001069.2	c.1072C>A	6	3154363	3154363	G	T	AD	De novo	LP	het.
SLC6A1	p.A288V	NM_003042.3	c.863C>T	3	11067472	11067472	C	T	AD	De novo	LP	het.
MECP2	p.Arg168Ter	NM_004992.3	c.502C>T	X	153296777	153296777	G	A	XL	De novo	P	het.
ALS2	p.K1174*	NM_020919.3	c.3520A>T	2	202588157	202588157	T	A	AR	Mat. and Pat. (HZ)	P	HZ
ACTG1	p.P70L	NM_001199954.2	c.209C>T	17	79479083	79479083	G	A	AD	De novo	P	het.
MAGEL2	p.N1084fs*22	NM_019066.4	c.3246delC	15	23889644	23889644		delG	AD	Unk., father unavailable	P	het.
AHDC1	p.R587fs*56	NM_001029882.3	c.1758dupA	1	27876869	27876869	dupT		AD	De novo	P	het.
ACAD9	p.P370fs*13	NM_014049.4	c.1109delC	3	128623308	128623308		delC	AR	De novo	P	cpd het.
ACAD9	p.R266W	NM_014049.4	c.796C>T	3	128618292	128618292	C	T	AR	Maternal	LP	cpd het.
AQP2	p.G64R	NM_000486.5	c.190G>A	12	50344803	50344803	G	A	AR	Mat. and Pat. (HZ)	P	HZ
HRAS	p.G12S	NM_176795.4	c.34G>A	11	534289	534289	C	T	AD	De novo	P	het.
ZC4H2	p.R198W	NM_018684.3	c.592C>T	X	64137746	64137746	G	A	XL	De novo	LP	hemiz.
BMP2	p.R170*	NM_001200.3	c.508C>T	20	6759053	6759053	C	T	AD	Maternal	P	het.
EHMT1		NM_024757.4	c.2712+1G>A	9	140695437	140695437	G	A	AD	De novo	P	het.
USP9X	p.P87fs*8	NM_001039590.2	c.260delC	X	40990727	40990727		delC	XL	De novo	P	het.
COL6A2	p.C37*	NM_001849.3	c.111C>A	21	47531501	47531501	C	A	AD	Paternal	LP	het.
SLC17A5	p.T178fs	NM_012434.5	c.533del	6	74348215	74348215		del	AR	Maternal	P	cpd het.
SLC17A5	p.R39C	NM_012434.5	c.115C>T	6	74354306	74354306	G	A	AR	Paternal	P	cpd het.
KMT2A	p.E2530Kfs*10	NM_005933.3	c.7588delG	11	118374204	118374204		delG	AD	De novo	P	het.
ARX	p.S319*	NM_139058.2	c.956C>A	X	25031156	25031156	G	T	XL	De novo	P	het.
ECEL1	p.F37fs*151	NM_004826.4	c.110_155del	2	233351209	233351254		del	AR	Mat. and Pat. (HZ)	P	HZ
KIFBP	p.L363fs*7	NM_015634.3	c.1086_1095delTCTTGATAAA	10	70775392	70775401		delTCTTGAT.	AR	Mat. and Pat. (HZ)	P	HZ
KIF1A	p.P305L	NM_001244008.1	c.914C>T	2	241715312	241715312	G	A	AD	De novo	P	het.
ZC4H2	p.R211W	NM_018684.4	c.631C>T	X	64137707	64137707	G	A	XL	Maternal	LP	hemiz.
NR3C2	p.Q919*	NM_000901.4	c.2767C>T	4	149035287	149035287	G	A	AD	Maternal	LP	het.
TBX5	p.E69*	NM_000192.3	c.205G>T	12	114839668	114839668	C	A	AD	De novo	P	het.
LAMA2	p.R1450*	NM_000426.3	c.4348C>T	6	129663524	129663524	C	T	AR	Maternal	P	cpd het.
LAMA2	p.D2383*	NM_000426.3	c.7144C>T	6	129785586	129785586	C	T	AR	Paternal	P	cpd het.
PMM2		NM_000303.2	c.174+1G>A	16	8895764	8895764	G	A	AR	Unk., father unavailable	P	cpd het.
PMM2	p.V60L	NM_000303.2	c.178G>T	16	8895767	8895767	G	T	AR	Maternal	LP	cpd het.
MBD5	p.P313fs*4	NM_018328.4	c.936dupA	2	149226448	149226448	dupA		AD	Unk., father unavailable	LP	het.
KMT2D	p.P498*	NM_003482.3	c.1491_1492delGC	12	49445974	49445975		delGC	AD	De novo	P	het.

NFIX	p.C102fs*17	NM_001271043.2	c.303dupC	19	13136086	13136086	dupC		AD	De novo	P	het.
CHD7	p.E871D	NM_017780.4	c.2613G>T	18	61729060	61729060	G	T	AD	Unk., father unavailable	LP	het.
HRAS	p.G13R	NM_005343.3	c.37G>C	11	534286	534286	C	G	AD	De novo	P	het.
SLC9A6	p.E356fs*11	NM_001042537.1	c.970_973delGAGT	X	135092671	135092674		delGAGT	XL	Maternal	P	het.
NSD1	p.I1122fs*3	NM_022455.4	c.3364dupA	5	176638764	176638764	dupA		AD	De novo	P	het.
OFD1	p.E829*	NM_003611.2	c.2364dupT	X	13779307	13779307	dupT		XL	Maternal	LP	hemiz.
WAC	p.S491fs*9	NM_016628.4	c.1335delG	10	28900749	28900749		delG	AD	De novo	P	het.
TUBA1A	p.K430_E434del	NM_006009.3	c.1288_1302delAAGGATTATG	12	49578847	49578861		delCTCCTCAT	AD	De novo	P	het.
CDK13	p.N842S	NM_003718.4	c.2525A>G	7	40085606	40085606	A	G	AD	De novo	P	het.
PTPN11	p.P491T	NM_002834.4	c.1471C>A	12	112926851	112926851	C	A	AD	Unk., father unavailable	LP	het.
DNAH9	p.R995fs*5	NM_001372.3	c.2984delG	17	11572742	11572742		delG	AR	Mat. and Pat. (HZ)	P	HZ
KMT2A	p.C1448Y	NM_001197104.1	c.4343G>A	11	118359339	118359339	G	A	AD	De novo	P	het.
LAMA1	p.Q2890*	NM_005559.3	c.8668C>T	18	6948444	6948444	G	A	AR	Maternal	LP	cpd het.
LAMA1	p.I136T	NM_005559.3	c.407T>C	18	7050874	7050874	A	G	AR	Paternal	VUS	cpd het.
AGA	p.T123Hfs*20	NM_000027.4	c.367_371delACACA	4	178360753	178360757		delTGTGT	AR	Paternal	P	cpd het.
AGA		NM_000027.4	c.911-2A>G	4	178354399	178354399	T	C	AR	Maternal	P	cpd het.
CHD3	p.R1025Q	NM_001005271.2	c.3074G>A	17	7803968	7803968	G	A	AD	De novo	LP	het.
FGFR3	p.Arg248Cys	NM_000142.4	c.742C>T	4	1803564	1803564	C	T	AD	De novo	P	het.
CTCF	p.H373Q	NM_006565.3	c.1119T>A	16	67654632	67654632	T	A	AD	De novo	LP	het.
SOX2	p.W166*	NM_003106.4	c.498G>A	3	181430646	181430646	G	A	AD	De novo	P	het.
ELN	p.G756fs	NM_000501.4	c.2262delA	7	73483117	73483117		delA	AD	Maternal	P	het.
NFIB	p.R89*	NM_001190737.2	c.265C>T	9	14307285	14307285	G	A	AD	De novo	P	het.
BCS1L	p.R56*	NM_001257342.2	c.166C>T	2	219525876	219525876	C	T	AR	Maternal	P	cpd het.
BCS1L	p.R90H	NM_001257342.2	c.269G>A	2	219525979	219525979	G	A	AR	Unk., father unavailable	LP	cpd het.
ANKRD11	p.E1282fs	NM_013275.5	c.3843dupT	16	89349107	89349107	dupA		AD	Paternal	P	het.
ANKRD11	p.Arg1466fs*87	NM_013275.5	c.4396_4397delAG	16	89348553	89348554		delCT	AD	De novo	P	het.
PUS7	p.L214fs	NM_019042.5	c.640_641delCT	7	105142956	105142957		delAG	AR	Maternal	LP	cpd het.
PUS7	p.S100fs	NM_019042.5	c.298_299delAG	7	105148661	105148662		delCT	AR	Unk., father unavailable	LP	cpd het.
EFTUD2	p.R354*	NM_004247.4	c.1060C>T	17	42945264	42945264	G	A	AD	De novo	P	het.
RIT1	p.F82L	NM_006912.6	c.246T>G	1	155874285	155874285	A	C	AD	De novo	P	het.
CDK13	p.N842S	NM_003718.5	c.2525A>G	7	40085606	40085606	A	G	AD	De novo	P	het.
SLC19A3	p. Val139Glu	NM_025243.4	c.416T>A	2	228564015	228564015	A	T	AR	Mat. and Pat. (HZ)	LP	HZ
AHDC1	p.K725fs*7	NM_001029882.3	c.217delA	1	27878410	27878410		delT	AD	De novo	P	het.
SIN3A	p.Q339*	NM_015477.2	c.1015C>T	15	75702621	75702621	G	A	AD	De novo	P	het.
KRAS	p.Thr74Pro	NM_033360.3	c.220A>C	12	25380238	25380238	T	G	AD	De novo	LP	het.
CHAMP1	p.E216_S217ins*	NM_032436.4	c.647_649dupAGT	13	115089964	115089966	dupAGT		AD	De novo	P	het.
IDUA	p.Gln70Ter	NM_000203.5	c.208C>T	4	981646	981646	C	T	AR	Mat. and Pat. (HZ)	P	HZ
COL2A1	p.Gly444Asp	NM_001844.5	c.1331G>A	12	48380895	48380895	C	T	AD	Maternal	LP	het.
KMT2D	p.K3258fs*72	NM_003482.3	c.9773delA	12	49431366	49431366		delT	AD	De novo	P	het.
POMT2	p.Arg659Gln	NM_013382.5	c.1976G>A	14	77745128	77745128	C	T	AR	Mat. and Pat. (HZ)	LP	HZ
FOXP2	p.Q196*	NM_014491.4	c.586C>T	7	114270049	114270049	C	T	AD	Unk., father unavailable	P	het.
PPP2R5D	p.Q200L	NM_006245.4	c.598G>A	6	42975009	42975009	G	A	AD	De novo	P	het.
RAD21	p.C585R	NM_006265.2	c.1753T>C	8	117859882	117859882	A	G	AD	De novo	P	het.
SYNGAP1	p.R248P	NM_006772.2	c.743G>C	6	33403371	33403371	G	C	AD	De novo	LP	het.
RPS6KA3	p.Y144*	NM_004586.2	c.432T>G	X	20212361	20212361	A	C	XL	De novo	P	het.
COL6A1	p.G284R	NM_001848.2	c.850G>A	21	47409043	47409043	G	A	AD	Paternal	P	het.
ARHGEF9	p.R289*	NM_015185.2	c.865C>T	X	62893977	62893977	G	A	XL	Maternal	P	hemiz.
PTPN11	p.I56V	NM_002834.4	c.166A>G	12	112888150	112888150	A	G	AD	Maternal	LP	het.
MAGEL2	p.Q93*	NM_019066.4	c.277C>T	15	23892613	23892613	G	A	AD	De novo	P	het.
PIEZO1	p.Pro1906Lysfs*55	NM_001142864.4	c.5716_5738del	16	88787087	88787109		del	AR	Paternal	LP	cpd het.
PIEZO1	p.Ile2270Thr	NM_001142864.4	c.6809T>C	16	88783084	88783084	A	G	AR	Maternal	VUS	cpd het.
MSL3	p.Q203*	NM_078629.4	c.607C>T	X	11780974	11780974	C	T	XL	De novo	P	het.
KAT6A	p.Gln1871*	NM_006766.3	c.5611C>T	8	41790127	41790127	G	A	AD	Unk., father unavailable	LP	het.
SHOC2	p.Met173Ile	NM_007373.3	c.519G>A	10	112724635	112724635	G	A	AD	De novo	LP	het.
KCNK4	p.A172E	NM_033310.3	c.515C>A	11	64064979	64064979	C	A	AD	De novo	LP	het.
MCPH1	p.T48I	NM_024596.3	c.143C>T	8	6272314	6272314	C	T	AR	Mat. and Pat. (HZ)	LP	HZ
MYRF	p.Ser264fs	NM_001127392.1	c.789dupC	11	61539020	61539020	dupC		AD	De novo	LP	het.
FGFR1	p.Arg254Trp)	NM_015850.3	c.760C>T	8	38282197	38282197	G	A	AD	De novo	LP	het.
GRIN2A	p.A818V	NM_000833.3	c.2453C>T	16	9862850	9862850	G	A	AD	De novo	LP	het.
PBX1	p.C273R	NM_002585.3	c.817T>C	1	164776894	164776894	T	C	AD	De novo	LP	het.
TRPV4	p.Phe471del	NM_021625.4(TRPV4)	c.1412_1414del	12	110232211	110232213		del	AD	De novo	P	het.
FOXC2	p.Glu343Ter	NM_005251.3	c.1027G>T	16	86601968	86601968	G	T	AD	Maternal	LP	het.
DHCR24		NM_014762.3	c.1218+1G>A	1	55319709	55319709	C	T	AR	Maternal	P	cpd het.
DHCR24	p.Gln402*	NM_014762.3	c.1204C>T	1	55319724	55319724	G	A	AR	Paternal	P	cpd het.
CSNK2B	p.Q31*	NM_001282385.1	c.91C>T	6	31635663	31635663			AD	Unk., mother unavailable	P	het.
SF3B4	p.Met1?	NM_005850.4	c.2T>C	1	149899650	149899650	A	G	AD	De novo	P	het.
ACTG2	p.Arg257His	NM_001615.4(ACTG2)	c.770G>A	2	74141963	74141963	G	A	AD	De novo	P	het.
PTPN11	p.Phe285Ser	NM_002834.4	c.854T>C	12	112915455	112915455	T	C	AD	De novo	P	het.
CACNA1A	p.I480fs	NM_001127221.1	c.1438delA	19	13428046	13428046		delT	AD	Maternal	LP	het.
SET	p.W226*	NM_001287737.1	c.678G>A	9	131456063	131456063	G	A	AD	De novo	P	het.
GRIN2D	p.V667I	NM_000836.2	c.1999G>A	19	48922979	48922979	G	A	AD	Unk., father unavailable	LP	het.
PTPN11	p.Phe285Ser	NM_002834.4	c.854T>C	12	112915455	112915455	T	C	AD	De novo	P	het.
PIEZO1	p.Met870Ile	NM_001142864.2	c.2610G>A	16	88799740	88799740	C	T	AD	Maternal	LP	het.
CDK10	p.W291*	NM_001160367.1	c.872G>A	16	89762102	89762102	G	A	AR	Mat. and Pat. (HZ)	P	HZ
CHD7	p.R494*	NM_017780.3	c.1480C>T	8	61655471	61655471	C	T	AD	Unk., father unavailable	P	het.
SRCAP	p.R2444Ter	NM_006662.3	c.7330C>T	16	30748691	30748691	C	T	AD		P	het.
ASH1L	p.R2044fs	NM_018489.2	c.6128dupT	1	155349883	155349883	dupA		AD	Unk., father unavailable	LP	het.
AR	p.Met746Thr	NM_000044.4(AR)	c.2237T>C	X	66937383	66937383	T	C	XL	Maternal	P	hemiz.
CEP55	p.Arg64*	NM_001127182.1	c.190C>T	10	95262876	95262876	C	T	AR	Maternal	LP	cpd het.

CEP55	p.His458Arg	NM_001127182.1	c.1373A>G	10	95287888	95287888	A	G	AR	Paternal	VUS	cpd het.
PTPN11	p.Asp61Gly	NM_002834.5	c.182A>G	12	112888166	112888166	A	G	AD	De novo	P	het.
SATB2	p.R389C	NM_001172509.1	c.1165C>T	2	200213432	200213432	G	A	AD	De novo	P	het.
SUZ12	p.Gly484fs	NM_015355.2	c.1451delG	17	30321596	30321596		delG	AD	De novo	LP	het.
FOXP3		NM_014009.3	c.648-2A>G	X	49112265	49112265	T	C	XL	Maternal	LP	hemiz.
SATB2	p.Q391P	NM_001172509.1	c.1172A>C	2	200213425	200213425	T	G	AD	De novo	LP	het.
SMAD4	p.I500V	NM_005359.5	c.1498A>G	18	48604676	48604676	A	G	AD	De novo	P	het.
KMT2D	p.Q2004*	NM_003482.3	c.6010C>T	12	49435971	49435971	G	A	AD	De novo	P	het.
IFT74		NM_025103.4	c.1685-1G>T	9	27062615	27062615	G	T	AR	Mat. and Pat. (HZ)	LP	HZ
HRAS	p.Gly12Asp	NM_005343.4	c.35G>A	11	534288	534288	C	T	AD	De novo	P	het.
CYP1B1	p.Glu387Lys	NM_000104.3	c.1159G>A	2	38298338	38298338	C	T	AR	Maternal	LP	cpd het.
CYP1B1	p.Arg368His	NM_000104.3	c.1103G>A	2	38298394	38298394	C	T	AR	Paternal	VUS	cpd het.
KMT2D	p.R5448*	NM_003482.3	c.16342C>T	12	49416133	49416133	G	A	AD	De novo	P	het.
CHD3	p.N1159L	NM_005852.4	c.3477C>A	17	7806361	7806361	C	A	AD	De novo	P	het.
SLC16A2	p.R197H	NM_006517.5	c.590G>A	X	73744208	73744208	G	A	XL	De novo	LP	het.
WDR45		NM_007075.3	c.235+1G>A	X	48935301	48935301	C	T	XL	De novo	P	het.
CHD7	p.Val1141fs	NM_017780.3	c.3422_3423delTG	8	61741265	61741266		delTG	AD	De novo	P	het.
ZBTB18	p.R45*	NM_006352.4	c.133C>T	1	244217236	244217236	C	T	AD	De novo	P	het.
SPTAN1	p.R139*	NM_001195532.1	c.415C>T	9	131337005	131337005	C	T	AD	De novo	LP	het.
ZFPM2	p.Arg117*	NM_12082.3	c.349C>T	8	106573638	106573638	C	T	AD	De novo	P	het.
CACNA1A	p.A446fs	NM_000068.3	c.1334_1335dupTA	19	13441071	13441072	dupTA		AD	Maternal	LP	het.
FGFR3	p.Arg248Cys	NM_000142.5	c.742C>T	4	1803564	1803564	C	T	AD	Unk., father unavailable	P	het.
PIEZO1	p.Val598Met	NM_001142864.2	c.1792G>A	16	88801339	88801339	C	T	AR	De novo	LP	cpd het.
RIT1	p.Ala94Ser	NM_006912.6	c.280G>T	1	155874251	155874251	C	A	AD	De novo	P	het.
TMEM237		NM_001044385.2	c.943+1G>T	2	202492798	202492798	C	A	AR	Paternal	P	cpd het.
TMEM237		NM_001044385.2	c.869+1delG	2	202493952	202493952		delC	AR	Maternal	P	cpd het.
FLNA	p.F2353fs	NM_001456.3	c.7035delT	X	153579374	153579374		delA	XL	De novo	LP	het.
HRAS	p.Gly13Asp	NM_005343.4	c.38G>A	11	534285	534285	C	T	AD	De novo	P	het.
CTCF		NM_006565.3	c.782-1G>C	16	67645853	67645853	G	C	AD	Unk., father unavailable	LP	het.
KAT6B	p.Pro1332fs	NM_012330.3	c.3995delC	10	76788577	76788577		delC	AD	De novo	P	het.
KMT2D	(p.Gln4157Ter	NM_003482.3	c.12469C>T	12	49426019	49426019	G	A	AD	De novo	P	het.
FGFR3	p.Lys652Glu	NM_000142.5	c.1954A>G	4	1807895	1807895	A	G	AD	De novo	P	het.
ARID1A	p.Glu59*	NM_006015.4	c.175G>T	1	27023069	27023069	G	T	AD	De novo	P	het.
COL1A1	p.Thr1431fs	NM_000088.3	c.4291delA	17	48262967	48262967		delT	AD	De novo	P	het.
HDAC8	p.T326_Pro332del	NM_018486.2	c.976_996delACACTATCCTCTC	X	71681863	71681883		delTGGGAT	XL	De novo	LP	het.
TRAF7	p.Arg371Q	NM_032271.2	c.1112G>A	16	2223814	2223814	G	A	AD		LP	het.
DYRK1A	p.R467*	NM_ 130438.2	c.1399C>T	21	38877745	38877745	C	T	AD	De novo	P	het.
ACTA1	p.Gly76Arg	NM_001100.3	c.226G>C	1	229568531	229568531	C	G	AD	De novo	P	het.
PTPN11	p.Asp61Gly	NM_002834.5	c.182A>G	12	112888166	112888166	A	G	AD	De novo	P	het.
PTPN11	p.Thr507Lys	NM_002834.4	c.1520C>A	12	112926900	112926900	C	A	AD	De novo	P	het.
CBL	p.Asp390V	NM_005188.3	c.1169A>T	11	119148949	119148949	A	T	AD	De novo	LP	het.
SUMF1				3				Del_exon9	AR	Mat. and Pat. (HZ)	LP	HZ
CNOT3	p.S415fs	NM_014516.3	c.1242dupC	19	54652230	54652230	dupC		AD	De novo	P	het.
KANSL1	p.L496fs	NM_015443	c.1485_1488delTCTT	17	44159852	44159855		delAAGA	AD	De novo	P	het.
FREM2	p.Thr251fs	NM_207361.4	c.750_751dupGA	13	39262231	39262232	dupGA		AR	Paternal	P	cpd het.
FREM2	p.Phe1722fs	NM_207361.4	c.5162dupA	13	39266643	39266643	dupA		AR	Maternal	P	cpd het.
DYNC2H1	p.Arg1423Cys	NM_001377.3	c.4267C>T	11	103029645	103029645	C	T	AR	Maternal	LP	cpd het.
DYNC2H1	p.Gln1573Pro	NM_001377.3	c.4718A>C	11	103036733	103036733	A	C	AR	Paternal	VUS	cpd het.
HBA2	p.Gly60Asp	NM_000517.6	c.179G>A	16	223207	223207	G	A	AR	Maternal	P	cpd het.
POC1A	p.R81Ter	NM_015426.5	c.241C>T	3	52183866	52183866	G	A	AR	Mat. and Pat. (HZ)	P	HZ
TUBA1A	p.Asn18Ser	NM_006009.4	c.53A>G	12	49580567	49580567	T	C	AD	De novo	P	het.
TPM1	p.Arg178His	NM_001018005.2	c.533G>A	15	63353108	63353108	G	A	AD	De novo	P	het.
COL2A1	p.G1362fs	NM_001844.4	c.4085delG	12	48368104	48368104		delC	AD	De novo	P	het.
POMT2	p.Gln733Ter	NM_013382.5	c.2197C>T	14	77743775	77743775	G	A	AR	Maternal	P	cpd het.
POMT2	p.Thr677Ile	NM_013382.5	c.2030C>T	14	77745074	77745074	G	A	AR	Paternal	VUS	cpd het.
DYNC2H1	p.Asp3015Gly	NM_001377.3	c.9044A>G	11	103091449	103091449	A	G	AR	De novo	P	cpd het.
DYNC2H1	p.Trp2155*	NM_001377.3	c.6464G>A	11	103052602	103052602	G	A	AR	Paternal	P	cpd het.
PIEZO1	p.Gln461*	NM_001142864.2	c.1381C>T	16	88802732	88802732	G	A	AD	Paternal	P	het.
HNRNPH2	p.Arg206Trp	NM_019597.5	c.616C>T	X	100667592	100667592	C	T	XL	De novo	P	het.
SHOC2	p.S2G	NM_007373.4	c.4A>G	10	112724120	112724120	A	G	AD	De novo	P	het.
ALPL	p.Tyr178His	NM_000478.4	c.532T>C	1	21890593	21890593	T	C	AR	Paternal	LP	cpd het.
ALPL		NM_000478.4	c.648+5G>C	1	21890714	21890714	G	C	AR	Maternal	VUS	cpd het.
SYNE1	p.Gln6319	NM_182961.3	c.18955C>T	6	152583184	152583184	G	A	AR	Maternal	P	cpd het.
SYNE1	p.Trp7761	NM_182961.3	c.23283G>A	6	152510405	152510405	C	T	AR	Paternal	P	cpd het.
ZEB2		NM_014795.3	c.917-1G>A	2	145157838	145157838	C	T	AD	De novo	P	het.
