# Supplementary material for: Diagnostic yield of pediatric and prenatal exome sequencing in a diverse population
Source: NPJ Genom Med. 2023 May 26;8:10. doi: 10.1038/s41525-023-00353-0 (PMC10220040; doi:10.1038/s41525-023-00353-0)
Supplement: Supplementary file 2 — Reporting Summary [file 41525_2023_353_MOESM2_ESM.pdf]

nature portfolio

Corresponding author(s): Anne Slavotinek
Last updated by author(s): Mar 23, 2023

Reporting Summary

Nature Portfolio wishes to improve the reproducibility of the work that we publish. This form provides structure for consistency and transparency in reporting. For further information on Nature Portfolio policies, see our Editorial Policies and the Editorial Policy Checklist.

Statistics

For all statistical analyses, confirm that the following items are present in the figure legend, table legend, main text, or Methods section.

n/a	Confirmed	
☒	☐	The exact sample size (*n*) for each experimental group/condition, given as a discrete number and unit of measurement
☒	☐	A statement on whether measurements were taken from distinct samples or whether the same sample was measured repeatedly
☐	☒	The statistical test(s) used AND whether they are one- or two-sided<br>*Only common tests should be described solely by name; describe more complex techniques in the Methods section.*
☐	☒	A description of all covariates tested
☐	☒	A description of any assumptions or corrections, such as tests of normality and adjustment for multiple comparisons
☒	☐	A full description of the statistical parameters including central tendency (e.g. means) or other basic estimates (e.g. regression coefficient) AND variation (e.g. standard deviation) or associated estimates of uncertainty (e.g. confidence intervals)
☒	☐	For null hypothesis testing, the test statistic (e.g. *F*, *t*, *r*) with confidence intervals, effect sizes, degrees of freedom and *P* value noted<br>*Give P values as exact values whenever suitable.*
☒	☐	For Bayesian analysis, information on the choice of priors and Markov chain Monte Carlo settings
☒	☐	For hierarchical and complex designs, identification of the appropriate level for tests and full reporting of outcomes
☒	☐	Estimates of effect sizes (e.g. Cohen's *d*, Pearson's *r*), indicating how they were calculated

Our web collection on statistics for biologists contains articles on many of the points above.

Software and code

Policy information about availability of computer code

Data collection	Provided in text; no custom codes or scripts were used
Data analysis	Provided in text

For manuscripts utilizing custom algorithms or software that are central to the research but not yet described in published literature, software must be made available to editors and reviewers. We strongly encourage code deposition in a community repository (e.g. GitHub). See the Nature Portfolio guidelines for submitting code & software for further information.

Data

Policy information about availability of data

All manuscripts must include a data availability statement. This statement should provide the following information, where applicable:

- Accession codes, unique identifiers, or web links for publicly available datasets
- A description of any restrictions on data availability
- For clinical datasets or third party data, please ensure that the statement adheres to our policy

Sequencing data has been uploaded to the Analysis Visualization and Informatics Lab-space (AnVIL) at the National Human Genome Research Institute. Clinical data is available from the authors on reasonable request. The sequencing was performed in a in a Clinical Laboratory Improvement Amendments (CLIA) licensed laboratory, the UCSF Clinical Cancer Genomics Laboratory (CLIA number is: 05D2034158).

Human research participants

Policy information about studies involving human research participants and Sex and Gender in Research.

Reporting on sex and gender	Patients of all sexes and gender were included
Population characteristics	Patients were enrolled at the University of California, San Francisco (UCSF) Benioff Children's Hospital Mission Bay and the Betty Irene Moore Women's Hospital. Pediatric patients were also enrolled at the Zuckerberg San Francisco General Hospital, UCSF Benioff Children's Hospital Oakland and the Community Medical Center in Fresno from August 2017 through April 2021. Prenatal patients were also recruited from collaborating groups across the country. Parental race and ethnicity information was obtained by self-report on a harmonized survey. URM pediatric and prenatal cases were defined as having at least one biological parent who self-identified as belonging to any non-white racial or ethnic minority group. If the information on one parent was missing, the child was considered URM if the responding parent was URM; if the responding parent was white or if information was missing for both parents, the self-identified race/ethnicity was considered unknown. Patients were defined as US if they fulfilled one or more of the following three criteria: (1) covered by MediCal health insurance (California's Medicaid option for low-income families), (2) living in a medically underserved area (MUA), as determined by the home zip code collected from the electronic medical record belonging to the patient and according to the Health Resources and Services Administration (HRSA) shortage designation criteria as listed on their website, and (3) living in a health professional shortage area (HPSA), as determined by the home zip code collected from the electronic medical record belonging to the patient, according to the HRSA shortage designation criteria.
Recruitment	We offered testing to patients seen in clinic for whom exome sequencing was clinically indicated, with a priority for US and URM families. Eligibility for pediatric and prenatal patients is described in Table S12. Pediatric patients were enrolled with the following indications: MCAs, DD/ID, metabolic disease, epilepsy, neurodegenerative disease/cerebral palsy (CP), and encephalopathy. Patients with MCA, metabolic disease, epilepsy, and neurodegenerative disease/CP were further categorized as having, or not having, ID. Prenatal eligibility criteria (Table S12) were based on imaging at the time of enrollment, and included one or more fetal structural abnormalities, an unexplained disorder of fetal growth, and one or more fetal effusions or non-immune hydrops. We supported the families with interpreting services and study staff who spoke Spanish. For the pediatric patients, the patient population seen at the Benioff Children's Hospitals in San Francisco and Oakland was diverse and we did not require specific community outreach efforts for patient recruitment.
Ethics oversight	The study was approved by the UCSF Institutional Review Board (IRB) (protocols 17-22504 and 17-22420), the Fresno Community Medical Center IRB (protocol 2019024), and was registered as two clinical trials ("Clinical Utility of Pediatric Whole Exome Sequencing", NCT03525431 and "Clinical Utility of Prenatal Whole Exome Sequencing", NCT03482141). Written informed consent was provided by adult participants >18 years of age, or by parents or legal guardians on behalf of their children <18 years of age or >18 years of age who were unable to consent independently. Assent was obtained from minors and intellectually disabled adults whenever possible.

Note that full information on the approval of the study protocol must also be provided in the manuscript.

Field-specific reporting

Please select the one below that is the best fit for your research. If you are not sure, read the appropriate sections before making your selection.

☒ Life sciences ☐ Behavioural & social sciences ☐ Ecological, evolutionary & environmental sciences

For a reference copy of the document with all sections, see nature.com/documents/nr-reporting-summary-flat.pdf

Life sciences study design

All studies must disclose on these points even when the disclosure is negative.

Sample size	Sample size was determined by the number of patients enrolled during the course of the study.
Data exclusions	There were no data exclusions in calculating the diagnostic yield.
Replication	We performed Sanger sequencing to verify variants identified on exome sequencing
Randomization	Subjects were not randomized.
Blinding	Investigators were not blinded.

Reporting for specific materials, systems and methods

We require information from authors about some types of materials, experimental systems and methods used in many studies. Here, indicate whether each material, system or method listed is relevant to your study. If you are not sure if a list item applies to your research, read the appropriate section before selecting a response.

Materials & experimental systems

n/a	Involved in the study
☒	☐ Antibodies
☒	☐ Eukaryotic cell lines
☒	☐ Palaeontology and archaeology
☒	☐ Animals and other organisms
☐	☒ Clinical data
☒	☐ Dual use research of concern

Methods

n/a	Involved in the study
☒	☐ ChIP-seq
☒	☐ Flow cytometry
☒	☐ MRI-based neuroimaging

Clinical data

Policy information about clinical studies

All manuscripts should comply with the ICMJE guidelines for publication of clinical research and a completed CONSORT checklist must be included with all submissions.

Clinical trial registration	The study was registered as two clinical trials (“Clinical Utility of Pediatric Whole Exome Sequencing”, NCT03525431 and “Clinical Utility of Prenatal Whole Exome Sequencing”, NCT03482141). The study was started on 8.1.2017 and completed on 5.13.2022.
Study protocol	Obtainable through UCSF IRB
Data collection	The study was started on 8.1.2017 and completed on 5.13.2022.
Outcomes	Outcome was the diagnostic yield of exome sequencing.
